# Inhibition of protein N-myristoylation blocks *Plasmodium falciparum* intraerythrocytic development, egress and invasion

**DOI:** 10.1371/journal.pbio.3001408

**Published:** 2021-10-25

**Authors:** Anja C. Schlott, Ellen Knuepfer, Judith L. Green, Philip Hobson, Aaron J. Borg, Julia Morales-Sanfrutos, Abigail J. Perrin, Catherine Maclachlan, Lucy M. Collinson, Ambrosius P. Snijders, Edward W. Tate, Anthony A. Holder

**Affiliations:** 1 Malaria Parasitology Laboratory, Francis Crick Institute, London, United Kingdom; 2 Molecular Sciences Research Hub, Imperial College, London, United Kingdom; 3 Department of Pathobiology and Population Sciences, The Royal Veterinary College, Hatfield, United Kingdom; 4 Flow Cytometry Science Technology Platform, Francis Crick Institute, London, United Kingdom; 5 Mass Spectrometry Proteomics Science Technology Platform, Francis Crick Institute, London, United Kingdom; 6 Malaria Biochemistry Laboratory, Francis Crick Institute, London, United Kingdom; 7 Electron Microscopy Science Technology Platform, Francis Crick Institute, London, United Kingdom; 8 Francis Crick Institute, London, United Kingdom; University of Melbourne, AUSTRALIA

## Abstract

We have combined chemical biology and genetic modification approaches to investigate the importance of protein myristoylation in the human malaria parasite, *Plasmodium falciparum*. Parasite treatment during schizogony in the last 10 to 15 hours of the erythrocytic cycle with IMP-1002, an inhibitor of *N*-myristoyl transferase (NMT), led to a significant blockade in parasite egress from the infected erythrocyte. Two rhoptry proteins were mislocalized in the cell, suggesting that rhoptry function is disrupted. We identified 16 NMT substrates for which myristoylation was significantly reduced by NMT inhibitor (NMTi) treatment, and, of these, 6 proteins were substantially reduced in abundance. In a viability screen, we showed that for 4 of these proteins replacement of the N-terminal glycine with alanine to prevent myristoylation had a substantial effect on parasite fitness. In detailed studies of one NMT substrate, glideosome-associated protein 45 (GAP45), loss of myristoylation had no impact on protein location or glideosome assembly, in contrast to the disruption caused by GAP45 gene deletion, but GAP45 myristoylation was essential for erythrocyte invasion. Therefore, there are at least 3 mechanisms by which inhibition of NMT can disrupt parasite development and growth: early in parasite development, leading to the inhibition of schizogony and formation of “pseudoschizonts,” which has been described previously; at the end of schizogony, with disruption of rhoptry formation, merozoite development and egress from the infected erythrocyte; and at invasion, when impairment of motor complex function prevents invasion of new erythrocytes. These results underline the importance of *P*. *falciparum* NMT as a drug target because of the pleiotropic effect of its inhibition.

## Abstract 2

Understanding the essential factors needed for malaria parasite development could help us find new therapeutic targets. This study reveals that N-myristoylation is a posttranslational modification of proteins essential for the parasites’ growth and their invasion of red blood cells.

## Introduction

The malarial parasite asexual blood stage is largely intraerythrocytic as the parasite invades, develops, and proliferates within red blood cells (RBCs) over a period of approximately 45 to 48 hours in the case of *Plasmodium falciparum*, the most lethal human parasite. Following invasion by the extracellular merozoite, the parasite profoundly modifies the RBC, growing through ring and trophozoite stages and then starting multiple rounds of nuclear division around 30 hours after invasion, resulting in schizont formation. Coincident with nuclear division, the parasite constructs a series of subcellular membranous structures that will form the inner membrane complex (IMC) and apical organelles such as the rhoptries and micronemes of the nascent 20 to 30 daughter merozoites. At the end of schizogony, the multinucleate coenocyte undergoes cytokinesis that draws the parasite plasma membrane (PM) around each of the developing progeny to form highly polarized merozoites, each with its own nucleus, a surface pellicle comprised of PM and IMC, and apical organelles for subsequent invasion and modification of a new RBC. Completion of this process is followed by lysis of the infected RBC and egress of the now extraerythrocytic merozoites, which attach to and invade new RBCs to establish the next intraerythrocytic proliferation cycle. This stage of the parasite life cycle is responsible for the disease pathology and therefore is a principal target for the development of drugs to kill the parasite.

Several parasite proteins synthesized during this cycle are modified by *N*-myristoyl transferase (NMT). This enzyme transfers the C14 fatty acid from myristoyl-CoA to the amino terminal glycine of substrate proteins, in a largely cotranslational event following the removal of the initiator methionine [1]. Substrate proteins have been predicted bioinformatically, using a partially conserved sequence recognition motif [2], or identified experimentally by metabolic incorporation of YnMyr, an alkyne-containing myristate analogue, which provides a convenient means to label such proteins and allow their purification and identification following the chemical addition of a biorthogonal tag [3–6]. Thirty-two *N*-myristoylated parasite proteins have been identified experimentally in the *P*. *falciparum* asexual blood stages (reviewed in [7]). These NMT substrates are targeted to membranous structures such as the PM and the secretory pathway, which has a key role not only in protein export but also in the biogenesis and function of the IMC and intracellular organelles as well as protein import into the apicoplast [7]. Other myristoylated proteins are targeted to the nucleus or exported to the host erythrocyte. They function in a diverse range of cellular pathways such as protein secretion, transport and homeostasis, ion channel regulation, and parasite motility, with their known enzymatic functions including kinase, phosphatase, and hydrolase activities [7]. About one-third of the experimentally identified NMT substrates were shown to be essential in parasite growth screens using insertional mutagenesis in *P*. *falciparum* [8] and gene knockout in *Plasmodium berghei* [9,10]; however, this genetic evidence fails to indicate whether or not *N*-myristoylation is essential for the proteins’ function.

NMT inhibitors (NMTis) have been developed that kill the parasite in vitro [11–13]. Each of these inhibitor classes has been shown to bind to the protein substrate binding site of NMT, and their mode of action was confirmed using a parasite expressing a variant NMT with an amino acid substitution that abolishes both inhibition of enzyme activity and inhibition of parasite growth [14]. One such inhibitor, IMP-1002, when added to a synchronous population of ring stage parasites, interrupts parasite development irreversibly during early schizont development (4 to 6 nuclei) and before the formation of the IMC, producing a parasite form that we have termed a “pseudoschizont” [3]. It is likely that this form results from inhibition of NMT in the trophozoite or early schizont stages. However, many NMT substrates are expressed abundantly later in schizogony and the consequence of NMT inhibition during this later stage, during a period of approximately 10 to 15 hours following commencement of nuclear division, is unknown. Protein myristoylation may result in increased membrane binding affinity; therefore, loss of myristoylation can cause aberrant subcellular targeting and consequent loss of protein function and even degradation [15]. At the cellular level, inhibition of schizont development, for example, through impaired nuclear division or defective formation of intracellular organelles, may prevent merozoite formation, parasite egress, merozoite invasion, and subsequent ring stage development. To investigate these potential phenotypes, we examined the effect of inhibitor added during schizogony on parasite development, egress, and invasion and on myristoylated protein location and stability. The results showed that NMTi treatment during schizogony did not stop nuclear division, but it did inhibit parasite development before egress. We then developed a genetic method to examine whether the N-terminal glycine (and hence myristoylation) of a selected set of 6 proteins was essential for parasite growth. For members of the chosen panel of NMT substrates, substitution of N-terminal glycine with alanine was detrimental to parasite growth. From this set of proteins, we focused on one glideosome-associated protein 45 (GAP45) to examine the importance of *N*-myristoylation for its localization and function. Induced replacement of the N-terminal glycine of GAP45 with alanine had no effect on protein targeting to the IMC, the protein’s palmitoylation, or egress, but it did prevent merozoite invasion. We conclude that protein myristoylation is important at different time periods for nuclear division, merozoite maturation prior to egress, and for RBC invasion, implying that NMTis impact multiple facets of parasite development and are therefore excellent leads for drug development.

## Results

To investigate the effect of an NMTi during schizogony, synchronized parasite populations were treated with either 140 nM IMP-1002 (the EC_90_ of the compound [14]) or DMSO during the period from 34 to 45 hours postinvasion (PI), after which the culture medium was exchanged to a drug-free medium at the first sign of parasite egress in the DMSO-treated culture. Parasite growth, invasiveness, and morphology were then assessed by flow cytometry of Hoechst-stained parasites and microscopy of Giemsa- or antibody-stained fixed parasites, while parasite proteomics were analyzed by mass spectrometry.

### Parasite proliferation is decreased significantly by inhibition of NMT during schizont differentiation, blocking parasite development before egress

**Fig 1:**
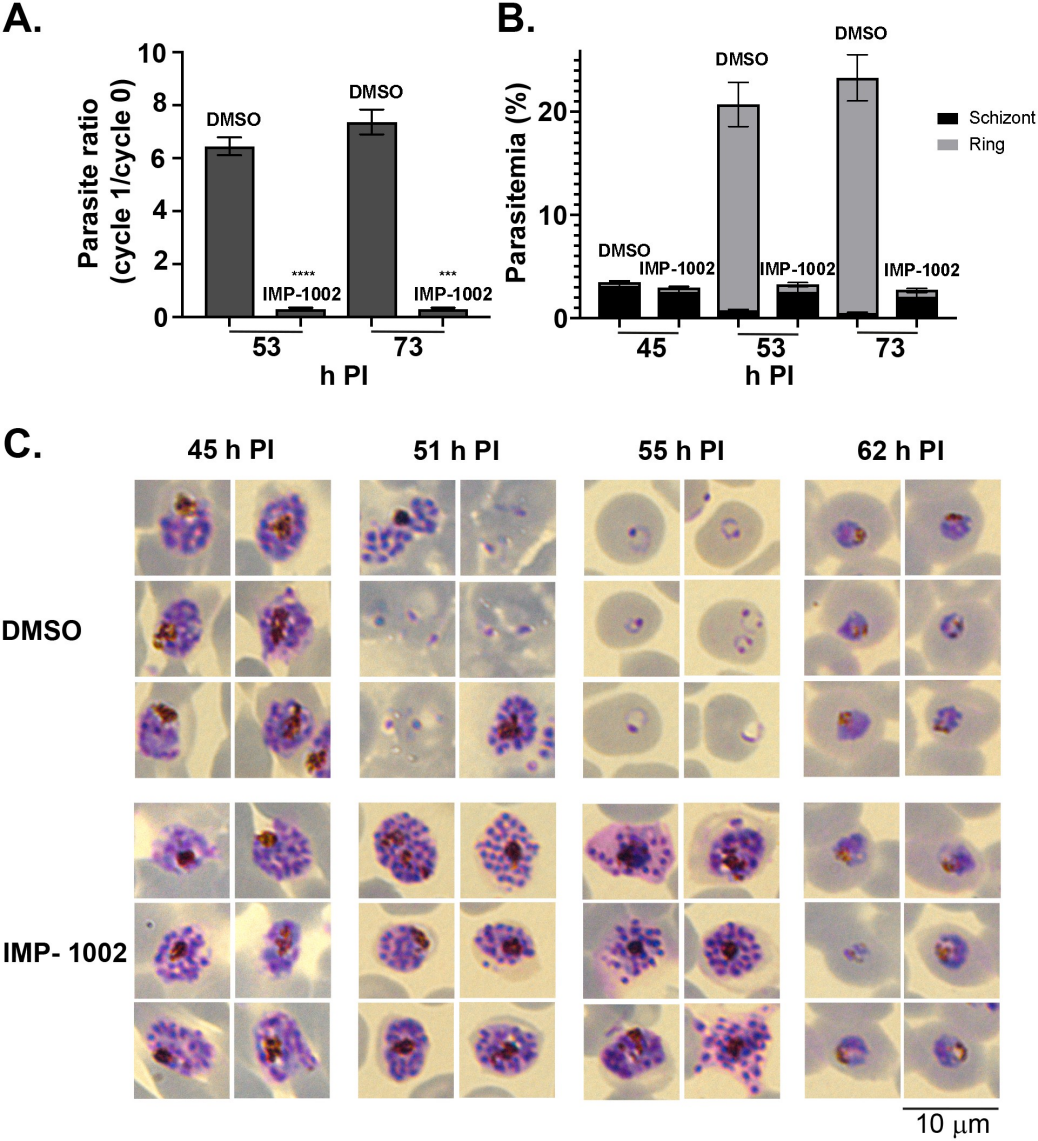
Inhibition of NMT during schizogony leads to a block in parasite development before merozoite egress from infected erythrocytes. **(A)** The parasite ratio (parasitemia of cycle 1/ parasitemia of cycle 0) at 53 and 73 h PI measured by flow cytometry. Equal numbers of IMP-1002- and DMSO-treated parasites, collected at 45 h PI and Percoll purified, were mixed with fresh erythrocytes for a growth assay. Data are from 2 biological replicates, each in triplicate (see S1 Data). The growth of IMP-1002–treated parasites was significantly lower (*p* < 0.0001 [****] for 53 h PI and *p* < 0.0003 [***] for 73 h PI: unpaired Student *t* test with Welch correction not assuming an equal SD, *n* = 3; 50,000 RBC counted per sample). **(B)** Percentage of schizonts and rings in the growth assay samples. While the number of schizonts remained the same during the period from 45 h PI to 73 h PI and few rings were detected even at 73 h PI in the IMP-1002 inhibitor–treated samples, the size of the schizont population dropped and the ring population increased substantially in the DMSO-treated control culture (data are from 2 biological replicates, each in triplicate; see S1 Data). **(C)** Giemsa staining to reveal parasite development and morphology after 11-hour drug treatment; 6 representative parasites are displayed for each time point and treatment. While there was no visible morphological difference between IMP-1002- and DMSO-treated schizonts at 45 h PI, by 55 h PI when most parasites in the control culture were ring stages, there was an irregular distribution of merozoites within the drug-treated schizonts. Parasites that survived drug treatment developed normally into trophozoites (shown at 62 h PI) (20 fields of view per sample, *n* = 3). Each panel width and the scale bar are 10 μm. h PI, hours postinfection; NMT, *N*-myristoyl transferase; RBC, red blood cell.

Flow cytometry analysis of samples stained with Hoechst dye demonstrated a significant drop in parasite proliferation resulting from IMP-1002 NMT inhibition compared with DMSO controls (*p* < 0.0001, S1 Fig). Therefore, both ring and schizont populations were examined separately to determine whether this decrease resulted from reduced parasite egress from infected erythrocytes or defective invasion into new erythrocytes. Using Percoll-purified schizonts, 2 samples, 8 hours (53 hours PI), and 28 hours (73 hours PI) after the start of egress of control (DMSO-treated) parasites were used to determine the growth rate (Fig 1A) and measure the schizont and ring stage parasitemia in each sample (Fig 1B). The growth rate dropped significantly in the presence of IMP-1002 compared with DMSO controls (*p* < 0.0001 for 53 hours PI and *p* < 0.0003 for 73 hours PI). In the DMSO-treated control culture, the schizont population decreased, and the ring population increased during the period between 45 and 73 hours PI, indicating merozoite egress and invasion, whereas the schizont population in IMP-1002–treated samples remained constant and few ring stage parasites were detected by 73 hours PI. These data indicate that NMT inhibition blocks parasite development in the schizont stage, before merozoite egress.

Giemsa staining and microscopy indicated that at 45 and 51 hours PI drug-treated schizonts looked similar morphologically to the DMSO-treated control parasites (Fig 1C). But at about 10 hours after the exchange to a drug-free medium (at 55 hours PI), IMP-1002–treated schizonts started to appear abnormal, and there was little evidence of invasion and new ring stage formation, suggesting that these parasites were not viable (Fig 1C). However, at 62 hours PI, some parasites that had escaped IMP-1002 NMT inhibition had developed into healthy-looking trophozoites. These results, obtained by light microscopy, complement those from the flow cytometry analysis and indicate that at IMP-1002 EC_90_, NMT inhibition blocks schizont development before merozoite egress in all but a small fraction of parasites.

### NMT inhibition changes substrate protein solubility and localization and may disrupt rhoptry biogenesis

NMT substrates may associate differently with membranes when parasites have been treated with NMTi. To examine this, schizonts were subjected to sequential solubility fractionation, and the distribution of specific proteins was revealed by western blotting. Both armadillo domain–containing rhoptry protein (ARO) and calcium-dependent protein kinase 1 (CDPK1), proteins that have an N-terminal myristoylation site and an adjacent potential palmitoylation site, were largely in the membrane-bound fraction prepared from DMSO-treated parasites (S2 Fig). However, following parasite treatment with IMP-1002, the proteins were either completely (in the case of ARO) or partially (in the case of CDPK1) found in the hypotonic buffer-soluble fraction. The IMC protein, GAP45, which has an N-terminal myristoylation site, an adjacent palmitoylation site, and an additional palmitoylation site near the carboxyl terminus [16], showed no difference in its solubility profile following IMP-1002 NMT inhibition. As controls, we identified the fractions enriched for cytoplasmic heat shock protein 70 (HSP70) and myosin tail interacting protein (MTIP), a component of the glideosome, formed together with GAP45 and other proteins. As expected, HSP70 was largely in the soluble fraction and MTIP was in the membrane bound fraction, and their behavior was not affected by IMP-1002 NMTi treatment of the parasite.

**Fig 2:**
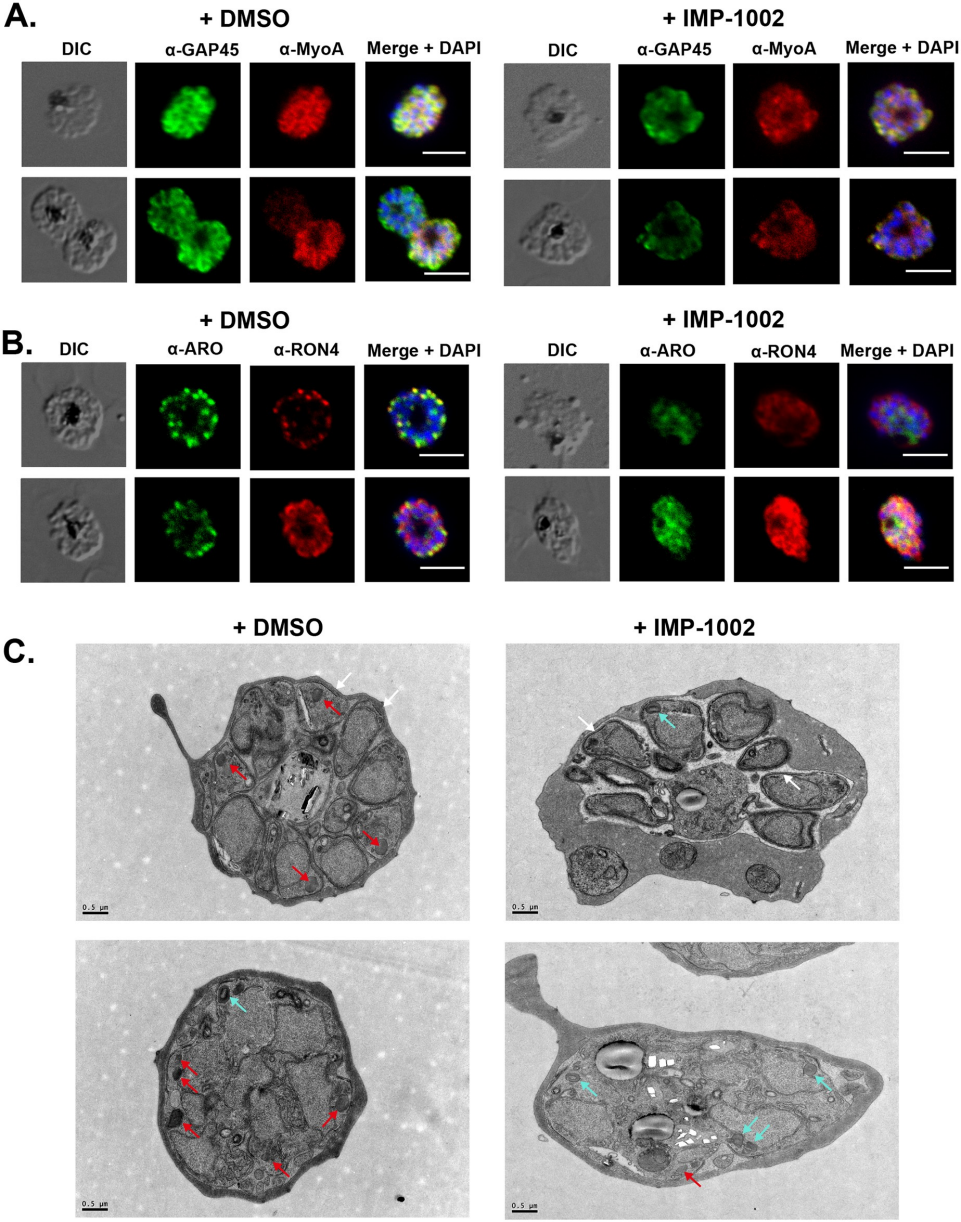
IMP-1002 treatment during schizogony has no effect on formation of the IMC but affects the subcellular location of ARO and RON4 and rhoptry biogenesis. Light microscopy images from indirect IFAs performed in duplicate on 3 separate occasions with fixed parasites from DMSO (control) and IMP-1002–treated parasites and protein-specific antibodies. Panels show the DIC image, the specific antibody location (green or red), and a merged image of antibody staining with DAPI staining of nuclei. Scale bar = 5 μm. **(A)** Two examples showing that the location of GAP45 and MyoA at the IMC appeared largely unchanged following drug treatment. **(B)** Two examples showing that the location of ARO and RON4 was affected by drug treatment, with the proteins appearing to be distributed throughout the cytoplasm of developing merozoites and a loss of distinct rhoptry staining in treated parasites. **(C)** Transmission electron micrographs of DMSO and IMP-1002–treated schizonts. IMC appears to develop normally in both parasite populations (white arrows). Two populations of rhoptry were evident: electron-dense structures (red arrows) that were predominantly in control cells and those with a lighter core or inclusion (cyan arrows) found largely in drug-treated cells. Scale bar = 0.5 μm. ARO, armadillo domain–containing rhoptry protein; DIC, differential interference contrast; GAP45, glideosome-associated protein 45; IFA, immunofluorescence assay; IMC, inner membrane complex; MyoA, myosin A; RON4, rhoptry neck protein 4.

By homology with *Toxoplasma gondii*, *N*-myristylated ARO is involved in the correct positioning of rhoptries at the apical end of developing merozoites [17], and, therefore, NMT inhibition may lead to defective or mislocalized rhoptries. To examine the effect of NMT inhibition on the subcellular protein location during schizont development, parasites were analyzed using an indirect immunofluorescence assay (IFA) with specific antibodies (Fig 2A and 2B). Following NMTi treatment, the location of IMC proteins, GAP45 and myosin A (MyoA) showed no discernible difference in their location and were present in both DMSO- and IMP-1002–treated cells (Fig 2A). ARO and rhoptry neck protein 4 (RON4) changed from being discrete to very diffuse in the cytoplasm of developing merozoites (Fig 2B).

Electron micrographs of schizonts from treated or untreated cultures revealed no gross ultrastructural changes resulting from NMT inhibition (Fig 2C), for example, formation of the IMC occurred. However, there was a distinct difference in rhoptries. Two populations were identified, those considered “normal” and predominant in the images from the control cells and those considered as “with a lighter core/inclusion,” which were more frequent in the images of inhibitor-treated cells. The frequencies of these 2 forms were quantified (in a blinded analysis of 40 images) and 32% (16/50) of the rhoptry structures in the inhibitor-treated schizonts had a lighter core compared to only 1.7% (1/59) of the rhoptry structures identified in the control schizonts. Examples of the 2 forms are indicated with red and cyan arrows, respectively, in Fig 2C.

These results indicate that schizont treatment with IMP-1002 can affect the membrane binding properties of some proteins and, for example, in the case of rhoptry proteins, may result in their mislocalization within the cell. Although there were no major morphological differences in the treated schizonts, analysis of the micrographs suggested that rhoptry biogenesis had been affected. We did not observe directly any significant change in rhoptry positioning in IMP-1002–treated cells, suggesting that the loss of N-myristoylation of ARO or other proteins does not significantly impact rhoptry positioning.

### IMP-1002 inhibits protein myristoylation and affects abundance of some NMT-substrates and non-myristoylated proteins

**Fig 3:**
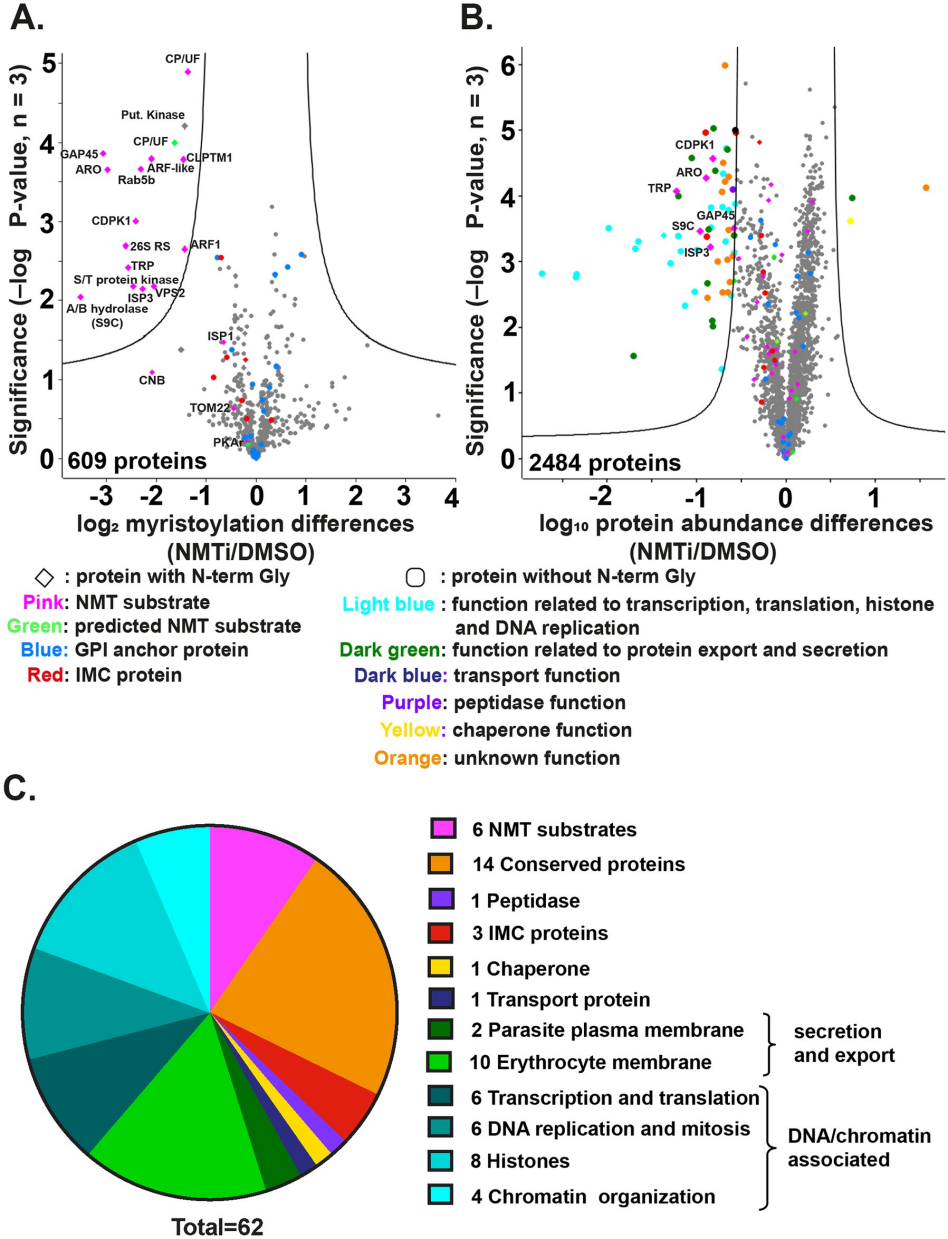
NMT inhibition changes the abundance of both myristoylated and non-myristoylated proteins. **(A)** Parasite proteins were metabolically labeled with YnMyr for 11 hours during schizogony, coupled to AzTB and enriched on Neutravidin coated agarose beads. LFQ analysis was used to measure the abundance of enriched proteins labeled in the presence or absence of IMP-1002. A 2-sample *t* test (permutation-based FDR, 250 permutations [number of randomizations], FDR 0.01, S0 = 0.5 [within groups variance]; *n* = 3 biological replicates, each with 3 technical replicates) revealed significant differences in myristoylated protein abundance between IMP-1002–treated and control (DMSO) samples. The lines on the graph indicate *t* test significance cutoff. The identity of some proteins is shown on the plot; symbols and color coding of individual proteins are explained below the plot. Full data are in S2 Data. **(B)** Quantitative whole proteome analysis using TMT protein labeling to measure protein abundance in parasite samples treated with either DMSO or 140 nM IMP-1002 and subsequent saponin lysis. A 2-sample *t* test (permutation-based FDR, 250 permutations, FDR 0.01, S0 = 0.8 [within groups variance], *n* = 3) revealed significant changes in overall protein abundance between the inhibitor-treated and control (DMSO treated) parasites. The identity of some proteins is shown on the plot; symbols and color coding of individual proteins are explained below the plot. Full data are in S3 Data. **(C)** Pie chart presentation of the 62 proteins significantly reduced in abundance following IMP-1002 treatment of schizonts from 34 to 45 h PI, with their associated grouping based on GO term analysis from PlasmoDB (Release 44, July 2019) The full listing is in S3 Data. FDR, false discovery rate; GPI, glycosyl phosphatidylinositol; h PI, hours postinfection; LFQ, label-free quantification; NMT, *N*-myristoyl transferase; NMTi, NMT inhibitor; TMT, tandem mass tag.

While the localization of NMT substrates can be studied by cellular fractionation or microscopy-based approaches, these methods provide no quantitative data on the effect of IMP-1002 inhibition on the modification or abundance of parasite proteins. Therefore, we used quantitative chemical proteomics to examine further the effect of NMT inhibition on both myristoylation of its substrates and the abundance of other proteins in the cell. The extent of myristoylation was studied using metabolic labeling with the myristic acid analogue YnMyr and label-free quantification (LFQ) by mass spectrometry to determine the relative abundance of individual myristoylated proteins in samples from parasites treated with either DMSO or IMP-1002 for 11 hours. Proteins were extracted and an AzTB biotin tag attached to the YnMyr-labeled proteins using click chemistry, then the tagged proteins were enriched by Neutravidin binding and elution. A total of 609 proteins were identified in the eluate from the Neutravidin-coated agarose beads (S2 Data). Sixteen NMT substrates showing a significant decrease in myristoylation in the presence of IMP-1002 were identified (Fig 3A and S2 Data). Fourteen of these 16 proteins were experimentally verified NMT substrates [3], while the 2 remaining proteins were a putative kinase (PF3D7_0321400) and a conserved protein homologue (PF3D7_0619700) of a Toxoplasma F-box protein [18] (Fig 3A). The modified N-terminal glycine was also identified for a number of NMT substrates, providing direct experimental evidence of myristoylation, for example, metal-dependent protein phosphatase 6 (PF3D7_1309200) and putative acylated pleckstrin-homology domain-containing protein (PF3D7_0414600) (S3 Fig). Glycosyl phosphatidylinositol (GPI) anchored proteins, which incorporate YnMyr through an ester linkage, and non-myristoylated IMC proteins were largely unchanged (analysis using adjusted *p*-values with a false discovery rate [FDR] of 0.01 and within group variance S_0_ = 0.5, *n* = 3).

To determine if there was an effect of NMT inhibition on overall protein abundance, the proteome of schizonts that had been incubated with or without 140 nM IMP-1002 from 34 to 48 hours PI was analyzed by mass spectrometry using tandem mass tag (TMT) labeling as a quantitative method, in combination with an additional high pH reverse fractionation step to increase coverage of the multiplex sample. A total of 2,484 proteins was identified (S3 Data), of which 62 were significantly reduced in abundance by IMP-1002 treatment (*t* test using an FDR cutoff of 0.01 and a within-group variance (S_0_) of 0.8) (Fig 3B and S4 Data). Gene ontology (GO) term analysis of these 62 proteins revealed 6 NMT substrates, together with several proteins involved in DNA replication and chromatin function, as well as a number of exported/secreted proteins (Fig 3C and S4 Data). The NMT substrates were ARO [GeneID: PF3D7_0414900], CDPK1 [PF3D7_0217500], GAP45 [PF3D7_1222700], alpha/beta hydrolase S9C [PF3D7_0403800], IMC sub-compartment protein 3 [ISP3; PF3D7_1460600], and tetratricopeptide repeat proteins [TRPs; PF3D7_0601600, PF3D7_0631000]. In previous studies, 3 of these proteins had been either suggested (CDPK1 and ARO) [8] or shown (GAP45 [19]) to be essential for growth of *P*. *falciparum* asexual blood stage parasites. Insertional mutagenesis of alpha/beta hydrolase S9C produces a slow growing phenotype [8] and *P*. *berghei* ISP3 is dispensable [20]. Of the TRP-encoding genes, PF3D7_0631000 was classified as essential, and PF3D7_0601600 was classified as dispensable in a recent *P*. *falciparum* mutagenesis screen [8]. However, none of these earlier studies addressed the essentiality of the N-myristoylation.

These data show that IMP-1002 treatment has a direct effect on the myristoylation of NMT substrates. Furthermore, there is a reduced abundance of both myristoylated and non-myristoylated proteins in the treated cells compared to those incubated with DMSO. There were 6 myristoylated proteins that were significantly reduced in abundance, suggesting that these NMT substrates are of particular importance. Therefore, we developed a genetic screen to look specifically at the importance of the N-terminal glycine of these proteins, and hence myristoylation, on parasite growth.

### A G2A/G2G CRISPR/Cas-9 screen identifies substrates for which myristoylation is required for parasite viability

The 6 NMT substrates significantly reduced in abundance by IMP-1002 treatment during schizogony were selected for further analysis to examine whether or not N-terminal myristoylation is essential for parasite growth. We developed a CRISPR/Cas-9 screen to determine the relative fitness of parasites following integration of a G2A codon or a G2G replacement codon at the myristoylation site for each of the 6 substrates. For TRP, we used PF3D7_0631000 for this screen, as it has been shown to be essential for parasite viability [8]. For each gene, Cas-9 was used to generate a double-strand break within the coding region close to the 5′ end of the gene using 2 different guides (S4 Fig and S1 Table), with repair mediated by plasmids containing either a G2A sequence to prevent myristoylation of the protein or a G2G sequence to allow it. Repair plasmids were mixed in equal proportion and added together to Cas-9/guide plasmids, linearized and used for parasite transfection. Then at the same time post-transfection, parasite genomic DNA was extracted, and integration-selective primers annealing to the inserted recodonized repair sequence were used to attach adapter sequences for Illumina sequencing (S2 Table). The ratio of G2A/G2G sequence reads for each parasite culture provides an indication of the relative viability of the G2G and G2A variants; with no fitness cost, a 1:1 ratio of the 2 forms would be expected in the parasite population.

**Fig 4:**
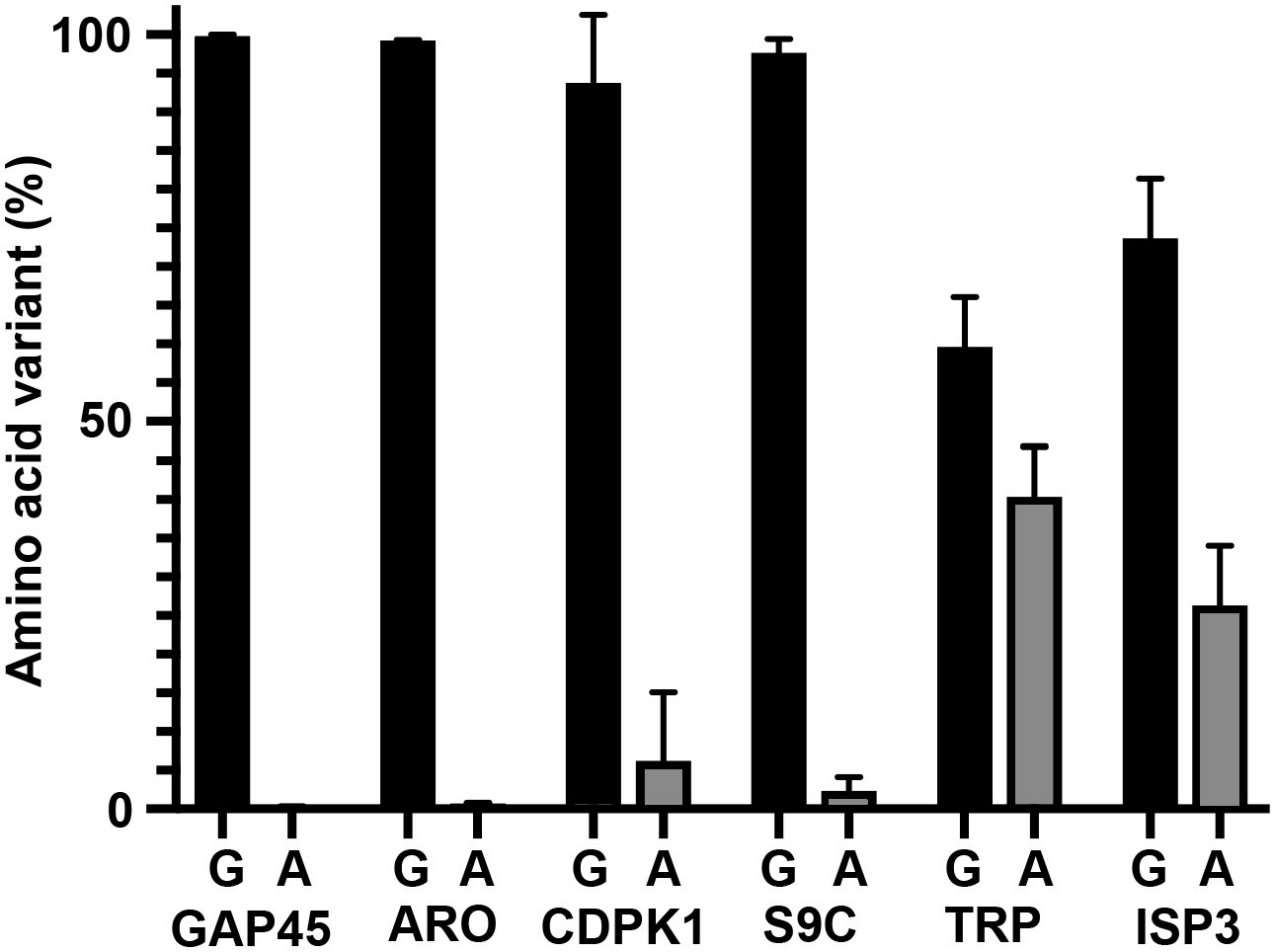
A viability screen of parasites containing either G2G or G2A at the myristoylation site in NMT substrates. A CRISPR/Cas-9 approach was used to insert G2A or G2G codons at the start of selected genes (see S4 Fig for details). Six genes were targeted using a 50:50 ratio of repair plasmids carrying a codon for G2G (Gly; silent mutation) or G2A (Ala, abolishing the myristoylation site). Illumina sequencing of products amplified using integration specific PCR primers was used to determine the distribution of each form in the parasite population following transfection (see S1 Data for the numbers). Each gene targeting was performed at least twice (*gap45*, *n* = 3) with 2 different guides (except for *aro* and *gap45* when 2 transfections with the same guide were performed). ARO, armadillo domain–containing rhoptry protein; CDPK1, calcium-dependent protein kinase 1; GAP45, glideosome-associated protein 45; ISP3, IMC sub-compartment protein 3; TRP, tetratricopeptide repeat protein.

For 4 of the 6 genes, almost 100% of the retrieved integrated sequences coded for an N-terminal glycine, suggesting that the N-terminal glycine is essential for these proteins (Fig 4), although the number of reads recovered for the CDPK1 gene was small (S4 Fig). For the TRP gene, only 60% of the reads were for the N-terminal glycine sequence and for ISP3, a 26% incorporation of the G2A variant was detected. Overall, the screen showed that for 4 out of the 6 NMT substrates, myristoylation is likely essential for viability (GAP45, ARO, CDPK1, and S9C), while for 2 substrates (ISP3 and TRP), myristoylation might be dispensable. To carry this analysis further, we focused on one protein, GAP45, which has been shown to be essential for motor complex formation and invasion, and used a genetics-based complementation approach to investigate the importance of GAP45 N-myristoylation.

### The N-terminal glycine of GAP45 is essential for parasite viability

We focused on GAP45, for which myristoylation appears to be essential for viability, for further detailed analysis of the consequence of lack of myristoylation. The strategy used a genetic complementation approach by further modification of an existing parasite that had been engineered to allow an inducible knockout of *gap45*. The *gap45* gene has been shown to be essential in the *gap45*:*ha3*:*loxP* parasite line (denoted as i[inducible]ΔGAP45), which has a loxPint intron after the first 49 base pairs, a loxP site after the stop codon, expresses HA-tagged GAP45, and allows an inducible knockout of the gene [19]. We inserted a second copy of the *gap45* gene together with its own promoter sequence into the *pfs47* locus to express either a wild-type (WT) GAP45 or GAP45[G2A] gene and examined whether or not this second gene complemented the induced knockout of *gap45*. Construction of these parasite lines is shown in S5 Fig. For both transfections, parasites were detected after 22 days and following confirmation of DNA integration, parasite lines were cloned by limiting dilution. The parasite clones used for complementation analysis are denoted as *gap45*:*ha3*:*loxP*::*comp_gap45[WT]* (c[complementary]GAP45[WT]) and *gap45*:*ha3*:*loxP*::*comp_gap45[G2A]* (cGAP45[G2A]), respectively.

**Fig 5:**
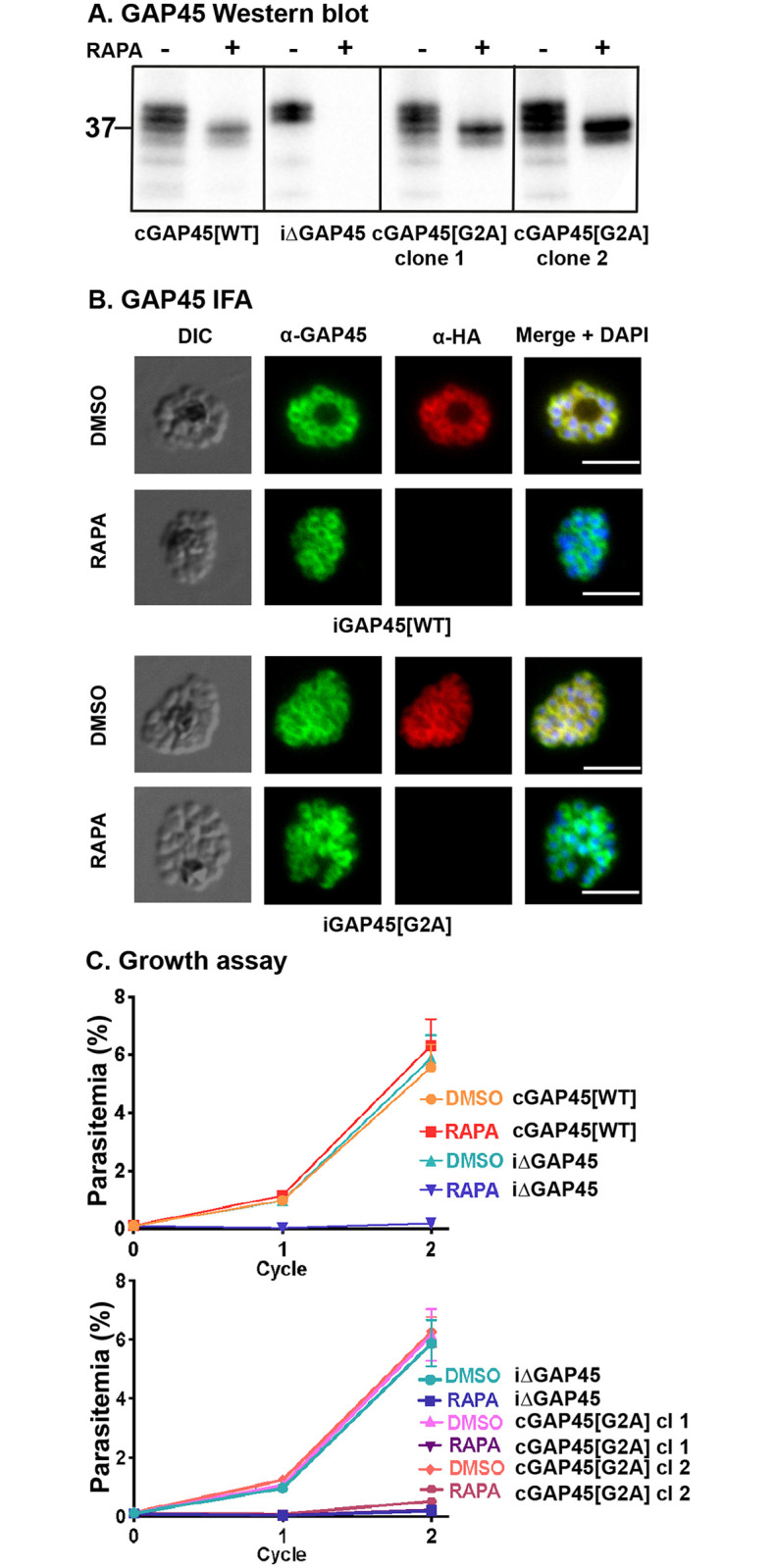
GAP45[G2A] is at the same subcellular location as GAP45, but the parasite has a growth defect. **(A)** Western blots showing successful rapamycin-inducible ablation of *gap45*:*ha3*:*loxP* expression (iΔGAP45), but expression of WT (cGAP45[WT]) and G2A (cGAP45[G2A]) from the Pfs47 site is unaffected. Note that HA3-tagged GAP45 adds additional mass to the protein resulting in a lower mobility in the gel. After rapamycin treatment, in GAP45[WT] and both GAP45[G2A] parasite clones the endogenous GAP45-HA3 is deleted but the second copy of the gene is still expressed (experiment performed in duplicate with 2 independent clones). Mobility of a molecular mass marker is indicated. **(B)** In the presence of rapamycin, the endogenous HA-tagged GAP45 protein is no longer present but GAP45 expressed from the second gene copy in the Pfs47 locus is located at the periphery of the developing intracellular merozoites, as judged by IFA. In the presence of DMSO, GAP45-HA3 is expressed at this subcellular location, as are GAP45[WT] and GAP45[G2A] in the presence or absence of rapamycin and DMSO for the *gap45*:*ha3*:*loxP*::*comp_gap45[WT]* (cGAP45[WT]) and *gap45*:*ha3*:*loxP*::*comp_gap45[G2A]* (cGAP45[G2A]) parasite clones, respectively (experiment performed in duplicate with 2 independent clones). Scale bar = 5 μm. **(C)** Growth of parasite lines following rapamycin or DMSO treatment over 2 cycles of development. Growth curves showing replication of the *gap45*:*ha3*:*loxP* (iΔGAP45) parasite line following rapamycin or DMSO treatment. Rapamycin induced excision of the *gap45*:*ha3*:*loxP* (iΔGAP45) locus produced parasites that were unable to replicate in vitro, a defect that can be complemented in the *gap45*:*ha3*:*loxP*::*comp_gap45[WT]* (cGAP45[WT]) but not in either of the 2 *gap45*:*ha3*:*loxP*::*comp_gap45[G2A]* (cGAP45[G2A]) clones. Data are from 3 biological replicates, each in triplicate with 2 independent clones (see S1 Data). Error bars show standard deviation. GAP45, glideosome-associated protein 45; IFA, immunofluorescence assay; WT, wild type.

First, we examined protein levels of the *gap45*:*ha3*:*loxP*, *gap45*:*ha3*:*loxP*::*comp_gap45[WT]* and *gap45*:*ha3*:*loxP*::*comp_gap45[G2A]* schizonts by western blotting and IFA using anti-GAP45 antibodies. The HA-tagged GAP45 is 4.3 kDa larger than GAP45 expressed at the same time from the second gene copy, which has no HA-tag [19], and, therefore, both predominant forms were visible on a western blot with anti-GAP45 antibodies (Fig 5A). Other fainter bands may reflect the phosphorylation of GAP45 [21]. In cycle 0, the cycle in which rapamycin treatment was given to induce gene excision (S6 Fig), expression of GAP45 in the *gap45*:*ha3*:*loxP* line was undetectable, whereas in the *gap45*:*ha3*:*loxP*::*comp_gap45[WT]* and *gap45*:*ha3*:*loxP*::*comp_gap45[G2A]* lines, GAP45 was present at approximately the same levels as in the DMSO-treated controls. The IFA analysis confirmed that rapamycin treatment abolished expression of the HA-tagged protein and that the gene inserted into the Pfs47 locus produces GAP45 that is indistinguishable in location from WT GAP45 (Fig 5B). By morphology, comparing the *gap45*:*ha3*:*loxP*, *gap45*:*ha3*:*loxP*::*comp_gap45[WT]* and *gap45*:*ha3*:*loxP*::*comp_gap45[G2A]*, these lines developed normally through cycle 0, confirming the previous observation that full-length GAP45 is not essential for schizont development [19]. This result also demonstrates that loss of N-terminal myristoylation in GAP45 does not alter the subcellular localization of this modified protein.

In subsequent cycles after rapamycin treatment, however, GAP45 expressed in the *gap45*:*ha3*:*loxP*::*comp_gap45[G2A]* integrant was not able to complement the *gap45*:*ha3*:*loxP* defect. After 2 cycles, neither rapamycin treated *gap45*:*ha3*:*loxP* nor *gap45*:*ha3*:*loxP*::*comp_gap45[G2A]* parasites were able to proliferate (Fig 5C). By contrast, the rapamycin treated *gap45*:*ha3*:*loxP*::*comp_gap45[WT]* parasites, and all 3 parasites treated with DMSO alone, continued to replicate. These data indicate that the N-terminal glycine and hence the myristoylation of GAP45 is indispensable for the survival of asexual blood stage parasites.

### GAP45[G2A] assembles into an intact glideosome and is *S*-palmitoylated but not *N*-myristoylated

With these 3 parasite lines, we were able to investigate the role of GAP45 in glideosome assembly and its posttranslational acylation. Previously, it had been shown that an N-terminal truncated GAP45, expressed together with WT GAP45, is incorporated into the glideosome [21], but in the absence of GAP45 the glideosome does not form [19]. In the *Plasmodium* glideosome model, based on the *T*. *gondii* assembly, the carboxyl-terminal domain of GAP45 interacts with MyoA and its light chains, MTIP and ELC, and binds to the IMC via GAP50, playing a role in motility and host cell invasion [22]. In the absence of GAP45, MTIP and MyoA are present at low levels and are not associated with the IMC (as assessed by IFA), although IMC formation is not impaired [19], leading to the conclusion that GAP45 is essential for correct motor complex assembly, but not for maintaining the structural integrity of the IMC in *Plasmodium* [19].

**Fig 6:**
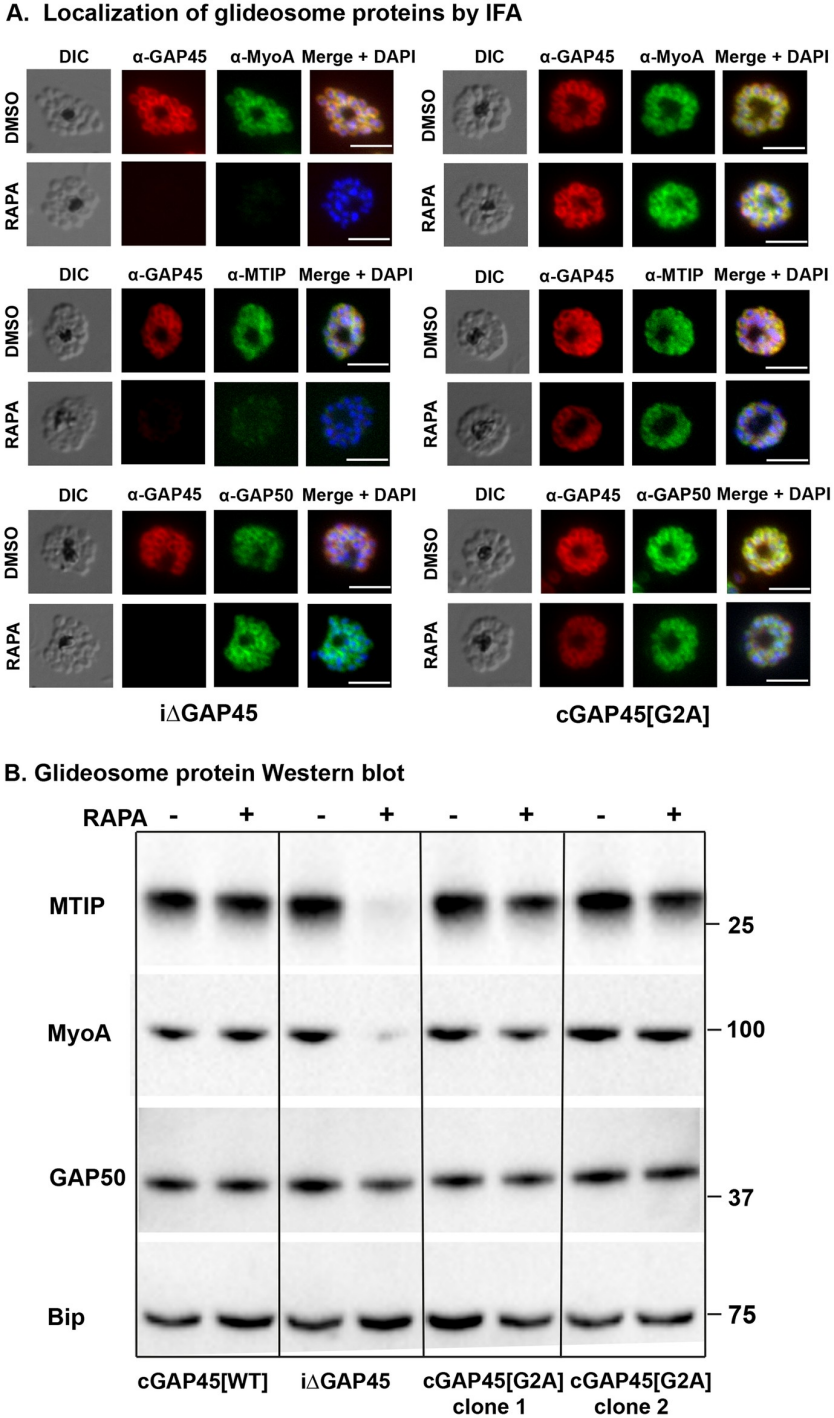
GAP45[G2A] parasites have a correctly localized glideosome. **(A)** GAP45[G2A] parasites show no defects in expression of the glideosome components MyoA and MTIP and which localized correctly at the periphery of merozoites. IFA showing the subcellular localization of GAP45-HA3, MyoA, MTIP and GAP50 in segmented schizonts of iΔGAP45 (*gap45*:*ha3*:*loxP*) and cGAP45[G2A] (*gap45*:*ha3*:*loxP*::*comp_gap45[G2A]*) in rapamycin and mock-treated (DMSO) parasites. Loss of GAP45 resulted in loss of detection of MTIP and MyoA at the IMC upon rapamycin treatment, while GAP45[G2A] is still able to recruit MTIP and MyoA. GAP50 staining is unchanged in both lines after rapamycin treatment (experiment performed in duplicate with 2 independent clones). Scale bar = 5 μm. **(B)** Deletion of the GAP45 gene results in loss of the glideosome proteins, MTIP and MyoA in the iΔGAP45 (*gap45*:*ha3*:*loxP*) line after rapamycin treatment, but they are retained in the cGAP45[WT] (*gap45*:*ha3*:*loxP*::*comp_gap45[WT]*) and cGAP45[G2A] (*gap45*:*ha3*:*loxP*::*comp_gap45[G2A]*) lines, as revealed by western blotting. GAP50, another glideosome protein and the ER protein, Bip are unaffected by the GAP45 gene deletion. Experiment performed in duplicate with 2 independent clones. Mobility of molecular mass markers is indicated. DIC, differential interference contrast; ER, endoplasmic reticulum; GAP45, glideosome-associated protein 45; IFA, immunofluorescence assay; MTIP, myosin tail interacting protein; MyoA, myosin A.

We examined the glideosome structure in *gap45*:*ha3*:*loxP*, *gap45*:*ha3*:*loxP*::*comp_gap45[WT]* and *gap45*:*ha3*:*loxP*::*comp_gap45[G2A]* parasites by IFA and western blot with or without rapamycin treatment. After rapamycin treatment of *gap45*:*ha3*:*loxP*::*comp_gap45[G2A]* parasites, both MyoA and MTIP proteins were detected in the correct location by IFA (Fig 6A) and at normal levels by western blot (Fig 6B), in contrast to the situation in *gap45*:*ha3*:*loxP* parasites where MyoA and MTIP were not detectable. The location and abundance of GAP50 were unaffected. These findings indicate that although GAP45 is important for recruiting MyoA and MTIP to the IMC, its N-terminal glycine and hence its myristoylation is not required for this activity.

**Fig 7:**
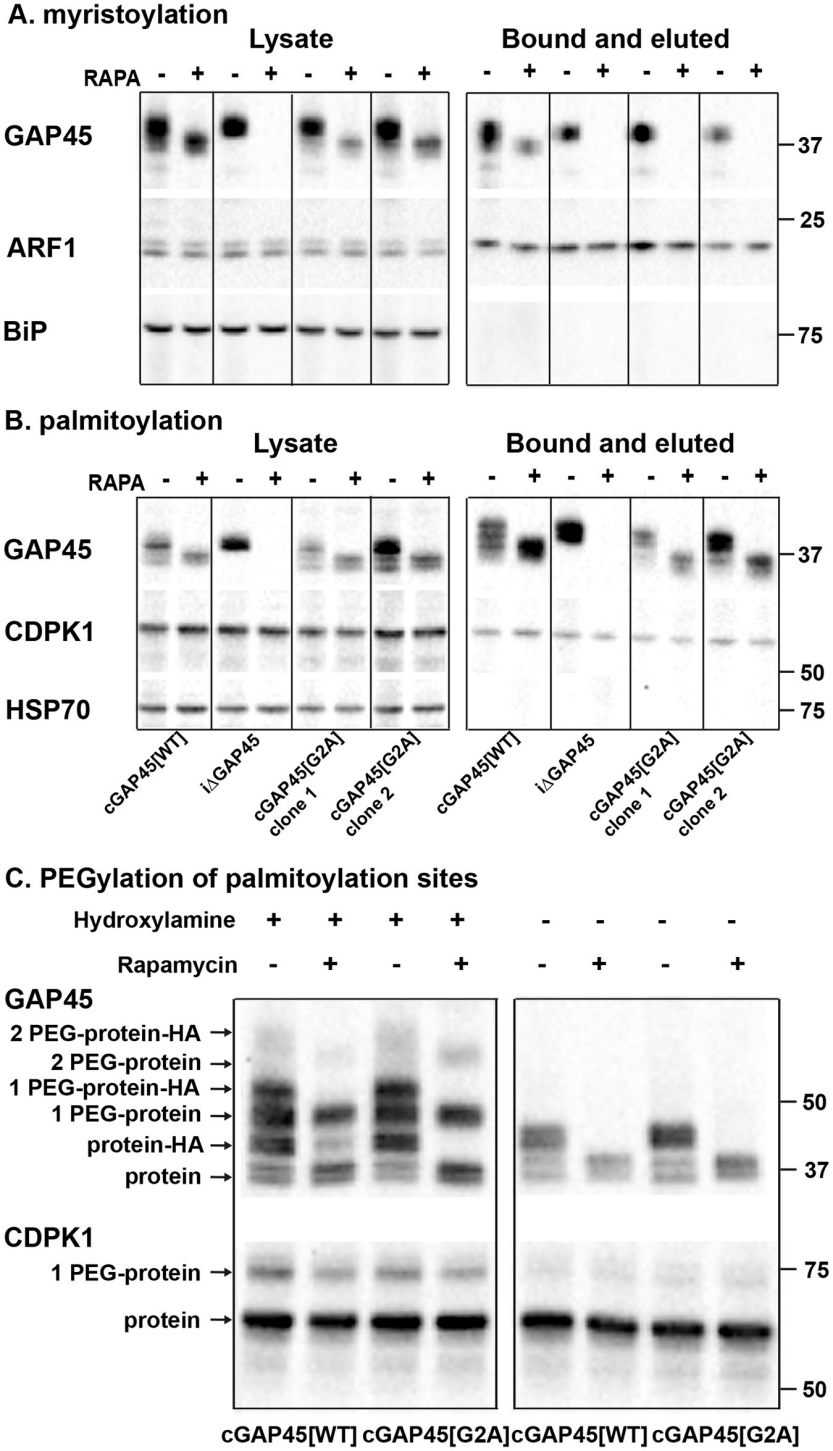
GAP45[G2A] is not myristoylated but is palmitoylated at a level similar to that of GAP45[WT]. Parasites were metabolically labeled in the presence or absence of rapamycin with either YnMyr (panel (A) myristoylation) or YnPal (panel (B) palmitoylation), then the capture reagent AzTB was attached using CuAAC (click)-chemistry and the labeled proteins were enriched on Neutravidin. **(A)** Western blot with anti-GAP45, anti-ARF1, and anti-BIP antibodies of enriched myristoylated proteins; a: lysate before enrichment, b: after enrichment: bound and eluted. **(B)** Western blots with anti-GAP45, anti-CDPK1, and anti-HSP70 antibodies of enriched palmitoylated proteins; a: lysate before enrichment, b: after enrichment: bound and eluted. After rapamycin treatment, only the *gap45*:*ha3*:*loxP*::*comp_gap45[WT]* (cGAP45[WT]) clone contained myristoylated GAP45. All other parasites showed no signal with the anti-GAP45 antibody after rapamycin treatment. The NMT substrate ARF1 was used as a positive control and was enriched from all 4 parasite clones, while the negative control BIP was not enriched as it is not myristoylated. All parasite clones except *gap45*:*ha3*:*loxP* (iΔGAP45) expressed enriched palmitoylated GAP45 after rapamycin treatment, indicating palmitoylation of GAP45[G2A]. CDPK1 was used as a positive control and was enriched for all 4 clones, while HSP70 was not enriched as it is not palmitoylated. The experiment was carried out with 2 independent clones of cGAP45[G2A] (gap45:ha3:loxP::comp_gap45[G2A]). **(C)** APE reveals site-specific S-fatty acid acylation of GAP45[G2A] at a similar level to that of GAP45. *gap45*:*ha3*:*loxP*::*comp_gap45[WT]* (cGAP45[WT]) and *gap45*:*ha3*:*loxP*::*comp_gap45[G2A]* (cGAP45[G2A]) parasites were treated with either rapamycin or DMSO and lysed at 48 h PI. Lysates were then subjected to APE, with or without hydroxylamine treatment to cleave esters, separated by SDS/PAGE, and analyzed by western blot with antibodies to either GAP45 or CDPK1. The mass of GAP45 is shifted by addition of the HA tag to the protein expressed from the endogenous locus, which is absent from the protein expressed from the *pfs47* locus. The number of PEGylation events is indicated. There was no evidence for a third palmitoylation of GAP45. CDPK1 was used a positive control; it has one palmitoylation site, visualized by the one PEGylation. Mobility of molecular mass markers is indicated. APE, acyl-PEG exchange; CDPK1, calcium-dependent protein kinase 1; CuAAC, copper(I)-catalyzed alkyne-azide cycloaddition; GAP45, glideosome-associated protein 45; h PI, hours postinfection; HSP70, heat shock protein 70; WT, wild-type.

To examine myristoylation, *gap45*:*ha3*:*loxP*::*comp_gap45[G2A]* (2 clones), *gap45*:*ha3*:*loxP* and *gap45*:*ha3*:*loxP*::*comp_gap45[WT]* parasites were treated with rapamycin, or DMSO, during cycle 0, metabolically labeled with YnMyr from 34 hours PI and harvested at 48 hours PI. Proteins were extracted and an AzTB biotin tag attached to the YnMyr-labeled proteins using click chemistry. Tagged proteins were enriched by Neutravidin binding and then analyzed by western blotting (Fig 7A). GAP45 was detected in the lysates and enriched protein fraction from all parasites treated with DMSO. However, after rapamycin treatment, only the *gap45*:*ha3*:*loxP*::*comp_gap45[WT]* parasite expressed the myristoylated protein. As a positive control for myristoylation and the enrichment procedure, the known NMT substrate, ADP-ribosylation factor (ARF1, PF3D7_1020900), was successfully enriched, the additional mass of YnMyr conjugated to AzTB resulting in a small mobility shift, whereas, as a negative control the non-myristoylated endoplasmic reticulum (ER) chaperone BiP (PF3D7_0917900), was not enriched. These results indicate that, as predicted, GAP45[G2A] is not myristoylated.

Since GAP45[G2A] is not myristoylated, its modification by palmitoylation was examined. GAP45 has 6 cysteines that are potential sites for this modification: 1 at the N-terminus (Cys5) and 5 close to the carboxyl terminus, of which 1 has been shown experimentally to be palmitoylated [16]. The 4 parasite lines were synchronized, rapamycin treated, and metabolically labeled with YnPal (heptadec-17-ynoic acid, also known as YnC14) to allow a biotin tag to be attached and the proteins enriched with Neutravidin coated beads. Samples were analyzed by western blot using anti-GAP45, anti-CDPK1 (CDPK1 has a single palmitoylation site; a positive control), and anti-HSP70 (used as a negative control as HSP70 has no palmitoylation site) (Fig 7B). GAP45 was present in all samples except those from rapamycin treated *gap45*:*ha3*:*loxP* parasites. CDPK1 was present in all fractions including the enriched palmitoylated sample, whereas HSP70 was absent from the palmitoylated protein fraction. These results indicate that GAP45[G2A] is palmitoylated, to a similar extent as GAP45[WT], and that this modification is independent of prior myristoylation of the protein.

Incorporation of YnPal provides no indication of the number of palmitoylated cysteines in individual proteins. Therefore, to examine how many cysteines are palmitoylated in the different GAP45 proteins, we used acyl-PEG exchange (APE) methodology [23]. Proteins in cell extracts were reduced, reactive cysteine residues capped with *N*-ethylmaleimide (NEM), and then acyl thioester bonds were cleaved with hydroxylamine treatment, to allow site-specific alkylation with a 10-kDa methoxy(polyethylene glycol)-maleimide (mPEG-Mal) mass-tag. Each tag addition to a former palmitoylation site results in a discrete mobility shift detected on a western blot with anti-GAP45 antibodies (Fig 7C). The samples were split after NEM treatment and then either treated with hydroxylamine, or left untreated to reveal the background of mPEG-Mal tagging. The *gap45*:*ha3*:*loxP*::*comp_gap45[G2A]* and *gap45*:*ha3*:*loxP*::*comp_gap45[WT]* parasites were used, with and without rapamycin treatment. In the absence of rapamycin, HA-tagged GAP45, GAP45[G2A], and GAP45[WT] were all tagged with mPEG-Mal, and after rapamycin treatment, only GAP45[G2A] and GAP45[WT] were labeled. The western blot suggested two 10-kDa band shifts, consistent with the addition of 2 mPEG-Mal moieties to both GAP45[WT] and GAP45[G2A], although the upper band was faint in both cases, and there was no evidence of a third site (Fig 7C). The intensity of each band quantified with ImageJ confirmed that the single palmitoylation species was the most abundant modified form of the protein (S3 Table). CDPK1 was used as a control protein and displayed a single mPEG-Mal shift, consistent with a single palmitoylation site (Fig 7C and S3 Table).

### Myristoylation of GAP45 is dispensable for egress but essential for RBC invasion

**Fig 8:**
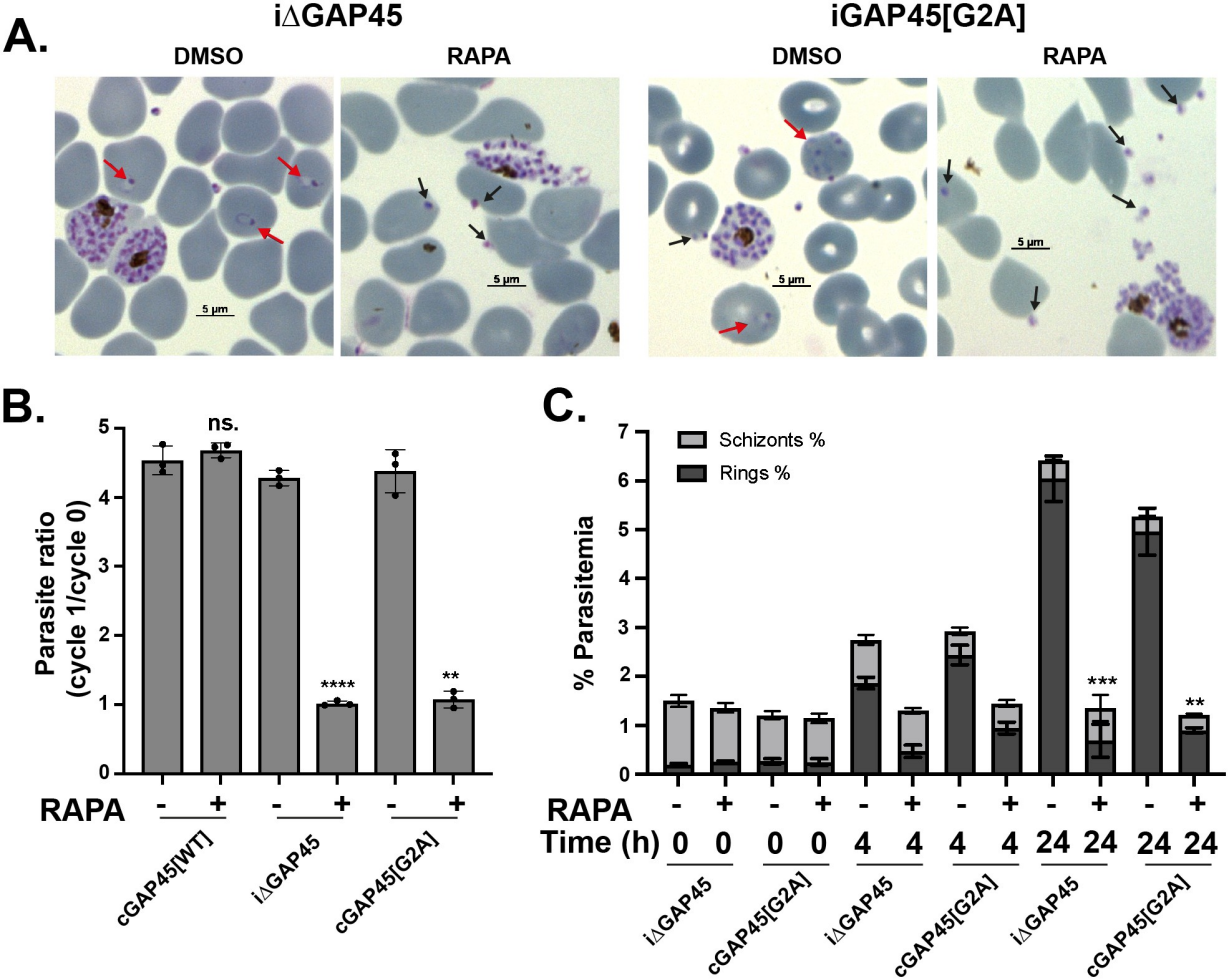
Parasites expressing GAP45[G2A] are not blocked at egress but show a defect in invasion. **(A)** Giemsa-stained thin blood smears of *gap45*:*ha3*:*loxP* (iΔGAP45) and *gap45*:*ha3*:*loxP*::*comp_gap45[G2A]* (cGAP45[G2A]) clones treated with DMSO or rapamycin, showing the substantial reduction in newly invaded ring stages after rapamycin treatment in both lines, while invasion occurs in the DMSO control (red arrows). Despite little invasion after rapamycin treatment, there is an abundance of extracellular merozoites visible with some apparently attached to erythrocytes (black arrows) indicating egress had occurred. **(B)** Invasion assay of *gap45*:*ha3*:*loxP*::*comp_gap45[WT]* (cGAP45[WT]), *gap45*:*ha3*:loxP (iΔGAP45), and *gap45*:*ha3*:*loxP*::*comp_gap45[G2A]* (cGAP45[G2A]) and showing the parasite ratio (parasitemia of cycle 1 / parasitemia of cycle 0) with and without rapamycin treatment. Data are from 3 biological replicates, each in triplicate (see S1 Data), Welch unpaired 2 tailed *p* < 0.0001 [****] for iΔGAP45, *p* = 0.001 [**] for cGAP45[G2A] and not significant [ns.] for cGAP45[WT]); error bars show standard deviation. **(C)** Percentage parasitemia of schizonts and rings in *gap45*:*ha3*:loxP (iΔGAP45) and *gap45*:*ha3*:*loxP*::*comp_gap45[G2A]* (cGAP45[G2A]) parasites at 0, 4, and 24 hours after culture of schizonts grown in the presence of DMSO or rapamycin. Data are from 3 biological replicates, each in triplicate with 2 independent clones (see S1 Data), Welch unpaired 2 tailed *t* test *p* < 0.0002 [***] for iΔGAP45 and *p* = 0.0042 [**] for cGAP45[G2A]); error bars show standard deviation. GAP45, glideosome-associated protein 45; WT, wild-type.

The growth assay over 2 generations indicated that *gap45*:*ha3*:*loxP*::*comp_gap45[G2A]* parasites had a severe growth defect (Fig 5), and previous work has shown that parasites lacking GAP45 are able to egress but not invade [19]. Therefore, the ability of *gap45*:*ha3*:*loxP*::*comp_gap45[G2A]* parasites to egress and invade after rapamycin treatment was investigated. Giemsa-stained thin blood smears of rapamycin treated *gap45*:*ha3*:*loxP*::*comp_gap45[G2A]* parasites at 48 hours PI revealed increased numbers of free merozoites and a lack of ring-stage parasites when compared to the DMSO-treated control (Fig 8A), a pattern similar to that observed with *gap45*:*ha3*:*loxP* parasites. An invasion assay was used to compare *gap45*:*ha3*:*loxP*::*comp_gap45[G2A]*, *gap45*:*ha3*:*loxP*::*comp_gap45[WT]* and *gap45*:*ha3*:*loxP* parasites, treated with either rapamycin or DMSO. *gap45*:*ha3*:*loxP*::*comp_gap45[WT]* parasites were able to invade erythrocytes normally after rapamycin treatment, but *gap45*:*ha3*:*loxP*::*comp_gap45[G2A]* and *gap45*:*ha3*:*loxP* showed significantly reduced invasion (parasitemia of cycle 1/parasitemia of cycle 0; *p* = 0.001 and *p* < 0.0001, respectively; Welch unpaired 2 tailed *t* test) (Fig 8B). In an assay with purified schizonts, schizont parasitemia had decreased after 24 hours when compared to the parasitemia at 0 or 4 hours, consistent with egress occurring normally (Fig 8C), but following rapamycin treatment both *gap45*:*ha3*:*loxP*::*comp_gap45[G2A]* and *gap45*:*ha3*:*loxP* parasite cultures contained significantly fewer new ring stages after 24 hours in the first cycle (Welch unpaired 2 tailed *t* test; *p* = 0.0042 for *gap45*:*ha3*:*loxP*::*comp_gap45[G2A]* and *p* < 0.0002 for *gap45*:*ha3*:*loxP*). These data indicate that myristoylation of GAP45 is necessary to generate the functional GAP45 that is essential for successful erythrocyte invasion.

## Discussion

The consequences of NMT inhibition with IMP-1002 for *P*. *falciparum* depend on the length of incubation with the inhibitor and its concentration, as well as the stage of parasite development to which the inhibitor is added. Over 30 different NMT substrates have been identified experimentally in the asexual erythrocytic stage, and just over 100 proteins of the total *Plasmodium* proteome are predicted to be myristoylated [3]. While many of the known NMT substrates likely have an essential function for the erythrocyte life cycle, the importance of *N*-myristoylation for that function is less clear, making it difficult to determine the key abnormalities resulting from NMT inhibition. One consequence of NMT inhibition for intraerythrocytic stages was the failure to assemble the IMC during early schizogony, leading to a block in development. This is likely due to the lack of myristoylation of IMC components such as GAP45, ISP1, and ISP3 resulting in the formation of pseudoschizonts—cells with only 4 to 5 nuclei that fail to undergo further karyogenesis and cytokinesis [3]. However, many proteins that are *N*-myristoylated are highly expressed during schizogony, in the last phase of intraerythrocytic development, and, therefore, we wished to examine in detail the consequence of treatment with NMTi specifically at this stage.

When IMP-1002 treatment was restricted to the last 11 hours of the cycle, parasites developed to schizonts that appeared morphologically fully mature, but parasite egress was prevented, leading to a significant drop in parasitemia in the subsequent cycle. Although there was no visible morphological change in parasites stained with Giemsa’s reagent, subcellular protein fractionation and immunofluorescence analysis showed that IMP-1002 treatment led to protein mislocalization. For example, the NMT substrate ARO is typically found in the membrane fraction, but, in the presence of IMP-1002, it was soluble. A similar change in membrane association was displayed by CDPK1, which is not a rhoptry protein but is attached to the PM. By contrast, GAP45 remained membrane bound, likely due to its additional *S*-palmitoylation close to the carboxyl terminus. Immunofluorescence images using antibodies to the rhoptry proteins, ARO and RON4, suggested that the localization of rhoptries was impaired by IMP-1002 treatment. By homology with *T*. *gondii* ARO, the *N*-myristoylated ARO is involved in the correct positioning of rhoptries at the apical end of developing merozoites [17], and, therefore, NMT inhibition might lead to defective or mislocalized rhoptries. Such a mislocalization of rhoptries has been observed after parasite treatment with 2-bromopalmitate (2-BP) [16], which is a highly promiscuous inhibitor of lipid metabolism, with impacts on *S*-acylation [24,25]. ARO has 2 cysteines (Cys5 and Cys6) that are likely palmitoylated [26], and, together with the N-terminal myristoylation, involved in membrane anchoring and rhoptry positioning. These findings support the idea that loss of myristoylation of ARO changes the localization of the protein. RON4 is not *N*-myristoylated but is contained within rhoptries, and its mislocalization is consistent with the whole organelle being affected. In contrast to the effect on the rhoptries, IMP-1002 treatment during schizogony appeared to have no gross effect on IMC formation. These data were extended and supported by the ultrastructural analysis of schizont morphology. No gross changes, for example, in IMC formation, were detected, but rhoptry structures could be distinguished into 2 forms, the characteristic electron-dense structure and a structure with a less dense core that was predominant in the IMP-1002–treated samples. We suggest that these morphological analyses support the idea that the drug interferes with the biogenesis of the rhoptry organelles.

The presence of IMP-1002 had a direct effect on protein *N*-myristoylation and abundance, as shown by 2 proteomic approaches that revealed differences in behavior of some substrates following NMT inhibition compared with the DMSO control. Using chemical proteomics to examine proteins modified by YnMyr, *N*-myristoylation of 16 proteins was significantly reduced during NMT inhibition compared with the DMSO-treated control. The YnMyr modified N-terminal peptide of several NMT substrates was also identified directly, including that of a metal-dependent protein phosphatase (PPM6) and a putative acylated pleckstrin-homology domain containing protein (APH), which had not been identified previously [3]. Due to the cotranslational nature of *N*-myristoylation, only those proteins synthesized during the YnMyr labeling window would have been purified and detected. Six of these substrates also showed a significant difference in protein abundance after IMP-1002 treatment compared with DMSO treatment, suggesting that NMT inhibition affects their overall stability. In addition to NMT substrates, other proteins were also decreased in abundance as a result of NMT inhibition. For example, IMP-1002 treatment led to a significant reduction of proteins involved in DNA chromatin organization and assembly, such as histones, and proteins involved in DNA replication, transcription, and protein translation. A second large group included secreted and exported proteins, especially some targeted to the erythrocyte membrane. The export of parasite proteins to the erythrocyte is maximal in early erythrocyte asexual stages, but some proteins are also expressed in schizonts, stored in the apical organelles and then transferred to the erythrocyte at invasion [27]. The observed changes in protein abundance may be due to a combination of altered transcription, protein synthesis, and protein degradation rates.

During intraerythrocytic development, protein synthesis starts to accelerate at around 18 to 24 hours PI [28], with the peak during schizogony. Total protein synthesis, as measured by metabolic incorporation of a methionine analogue, is not directly affected by NMTi [3], consistent with a selectivity and mode of action distinct from a direct effect on translation. However, since *N*-myristoylation is a cotranslational process, it is possible that NMTi and protein synthesis inhibition may be connected through common essential downstream factors or pathways. In the whole proteome analysis, proteins involved in DNA synthesis, transcription, translation, and chromatin organization were less abundant following IMP-1002 NMT inhibition compared with DMSO treatment, strengthening the hypothesis of a connection between DNA and protein synthesis and NMT inhibition. NMT inhibition may also delay parasite development, even though there was no observed effect on nuclear division, slowing down essential processes during schizogony and leading to changes in protein abundance.

In addition to causing protein mislocalization as observed for ARO, inhibition of NMT may also result in the misfolding of its substrates, leading to their degradation and reduced abundance. For example, NMT inhibition leads to death through apoptosis of several cancerous cell types, potentially as a result of ER stress and an unfolded protein response [29]. Inhibition of NMT may also lead to an imbalance in favor of other N-terminal protein modifications (NPMs) such as *N*-α-acetylation (NAT) carried out by N-terminal acetyltransferase [30] or N-terminal ubiquitination [15,31]. Usually, these NPMs occur cotranslationally through ribosome-associated protein biogenesis factors (RPBs) that interact with the ribosome and show a degree of competition in their binding [32]. For example, there is some indication of competition between NAT and *N*-myristoylation [2,33]. Additional experiments are necessary to investigate this further. In summary, treatment with NMTi results in mislocalization and reduced abundance of certain substrates that together may be responsible for the observed phenotype. An NMTi used to study myristoylation has an effect on many substrates simultaneously, and the phenotype reflects the resultant pleiotropic consequences.

Because it is difficult to draw conclusions as to which of the myristoylation provides the greatest contribution to the observed phenotypes, we supplemented the inhibitor studies with genetic approaches. We expected that proteins with the greatest reduction in YnMyr labeling and abundance are those most affected by NMT inhibition, and these might contribute most to the observed phenotypes. In order to study *N*-myristoylation of particular substrates in isolation, the proteomic data sets were used to select 6 NMT substrates for a G2A substitution screen to determine the essentiality of the N-terminal glycine. To address individually the importance of myristoylation for these substrates, we developed a competition screen to study parasite viability following the integration of N-terminal glycine or N-terminal alanine constructs to repair a double strand break induced by CRISPR/Cas-9. This screen showed that for 4 out of 6 substrates the parasites preferentially incorporated the glycine codon at the second position with a ratio of greater than 80% and for ARO, GAP45, and S9C nearly 100%. This strongly suggests the essentiality of myristoylation for at least 4 of the tested substrates. This approach does not allow phenotypic characterization of parasites lacking the N-terminal glycine in a specific substrate; therefore, GAP45 was selected for further analysis using a gene complementation approach to study the phenotype that results from the lack of myristoylation of this NMT substrate.

GAP45 is essential for glideosome assembly and erythrocyte invasion, as shown recently by using an inducible DiCre system to knockout the gene [19]. To complement this knockout, we placed the GAP45 gene in the Pfs47 gene locus, using 2 forms of the gene: one in which the second codon of the open reading frame encoded glycine, and a second in which the second codon encoded alanine. Interestingly, in both these constructs GAP45 appeared to be correctly targeted within the cell and allowed assembly of the glideosome as indicated by the correct location of MyoA and MTIP, which are not present in the absence of the complementing GAP45 gene copy. Although only the WT and not the G2A protein was myristoylated, both the G2A and WT GAP45 proteins were palmitoylated to a similar extent. Therefore, the single point mutation in the GAP45 gene, resulting in the presence or absence of the N-terminal myristoylated glycine, had a profound effect on parasite invasion, indicating that GAP45 myristoylation is essential for the function of the motor in invasion but not for motor assembly. It is possible that a low affinity interaction between GAP45 and the PM is essential, but to facilitate the dynamic changes that may be necessary for motor function (such as the passage along the membrane of the moving junction between parasite and RBC), the strength of the interaction needs to be modulated by differential palmitoylation/depalmitoylation of the cysteine close to the N-terminus or by GAP45 interaction with other proteins. The requirement for GAP45 myristoylation in invasion is clear, but further work is needed to clarify the importance of further mechanisms. For example, the role of Cys5 palmitoylation should be addressed in future experiments to investigate the necessity of a second modification of the protein to complement myristoylation for dynamic membrane binding [34].

In conclusion, using small molecule inhibitors of NMT and genetic methods to replace the N-terminal glycine in NMT substrates, we have shown the importance of these substrates and their myristoylation at different stages in parasite development. As a consequence of these multiple effects, inhibitors targeting NMT provide outstanding antimalarial parasite activity.

## Materials and methods

### Parasite culture

*P*. *falciparum* 3D7 parasites were cultured in vitro in RPMI 1640 medium containing 0.5% (w/v) Albumax II at 2% to 5% hematocrit as described [35]. Parasites cultures were gassed with 90% N_2_, 5% CO_2_, and 5% O_2_ and incubated at 37°C. Parasites were synchronized using 70% Percoll gradients to purify schizont stages, with a subsequent reinvasion followed by sorbitol treatment as described [36].

### Determination of parasitemia by flow cytometry

Synchronized parasites were incubated with DMSO or IMP-1002 and samples were fixed in 4% paraformaldehyde (PFA), 0.2% glutaraldehyde for 1 hour at 45 hours PI. Then, samples were washed in PBS and labeled with 1:500 of 10 mg/mL Hoechst 33342 (New England Biolabs, Hitchin, United Kingdom Cat# 4082S) for 10 minutes with a subsequent wash in PBS. For flow cytometry analysis, a BD CL1 Fortessa D Analyzer or Aria Fusion Sorter and Analyzer with FACSDiva software v8.0.1 were used with the 450–50 filter, counting 50,000 RBCs per sample. Data were analyzed using FlowJo LLC 2006–2015. Gating for RBCs was achieved by plots of forward scatter area against side scatter area (gate = P1). Doublet discrimination required gating on a plot of forward scatter height against forward scatter width (gate = P2) followed by a plot of side scatter height against side scatter width (gate = P3). A Hoechst-stained uninfected RBC sample was used as a negative control to gate on the infected population only on a forward scanner area against UVA fluorescence with 450–50 standard filter (gate = P4). Parasitemia was determined by the number of cells identified in gate P4 as a percentage of those in gate P3. The median fluorescence intensity (MFI) of each sample was used to determine the median number of nuclei per sample by normalizing it to the MFI of a control sample containing synchronized rings with a known MFI corresponding to one nucleus. Biological replicates used RBCs from different individual blood donors.

### Subcellular fractionation

Parasites were subjected to sequential fractionation to determine the solubility of proteins, using a method described previously [37]. Schizont proteins were fractionated by sequential solubilization using hypotonic and high salt buffers to release soluble cytosolic proteins, followed by a high pH sodium carbonate extraction to solubilize peripheral membrane proteins (carbonate soluble) but not tightly associated membrane proteins such as integral membrane proteins (carbonate insoluble). The distribution of specific proteins in the different fractions was revealed by western blotting.

### Western blot analysis

Proteins separated by SDS-PAGE were transferred to nitrocellulose membrane using the iBLOT Transfer system (Thermo Fisher Scientific United Kingdom). Following blocking overnight at 4°C in 5% (w/v) dried milk, 0.05% (v/v) Tween20 in PBS (PBS-T), membranes were incubated with primary antibody for 1 hour at room temperature (RT) in 5% milk in PBS-T, followed by three 5-minute washes in PBS-T and a subsequent incubation with species-specific secondary antibody (goat-anti-rabbit/rat/mouse IgG-HRP, Invitrogen, United Kingdom 1:2,500) for 1 hour in 5% milk in PBS-T. After a final three 5-minute washes, the membrane was incubated with either 1 ml of Amersham ECL substrate western blotting detection reagent (GE Healthcare, Chalfont St Giles, United Kingdom lot # 9622301) or for higher sensitivity, BioRad Clarity Western ECL substrate (BioRad, Watford, United Kingdom Cat # 170–5060), used according to the manufacturers’ instructions. The signal was visualized on a BioRad ChemiDoc MP Imaging System.

### Indirect IFA

For IFA, thin smears of parasitized RBC on slides were air dried, fixed in 4% PFA in PBS for 10 to 20 minutes, permeabilized in 0.1% (v/v) Triton X-100 in PBS for 10 minutes, and blocked with 3% bovine serum albumin (BSA) in PBS for at least 30 minutes at 4°C. Slides were then probed with the appropriate dilution of primary antibody in a humidified chamber at RT for 1 hour before being washed 3 times in PBS. Secondary antibody conjugated with Alexa Fluor 488 or 594 was added for 1 hour at the appropriate dilution, followed by 3 washes in PBS. Slides were mounted in ProLong Gold Antifade mounting medium containing DAPI (4′,6-diamidino-2-phenylindole) and viewed on a Nikon Eclipse Ni-E imaging system with a Hamamatsu Orca-flash 4.0 digital camera and a Plan apo λ 100×/1.45 oil immersion objective. Images were captured using Nikon NIS-Elements software, generating Z-stack images of individual parasites, using deconvolution options and exporting the image as a tiff file. Alternatively, images were processed using Fiji software [38]. Identical exposure conditions were used for each wavelength in treated (rapamycin or IMP-1002) and control (DMSO) samples.

### Electron microscopy

Samples were fixed by adding an equal volume of pre-warmed (37°C) double strength fixative (8% PFA in 0.2 M phosphate buffer [PB] at pH 7.4) for 15 minutes at 37°C, centrifuged (250 *g* for 2 minutes) and the pellet resuspended in single strength fixative (4% PFA and 2.5% glutaraldehyde in 0.1 M PB at pH 7.4) for 30 minutes at RT. The sample was centrifuged again (250 *g* for 2 minutes), and the pellet resuspended in 0.1 M PB for 10 minutes at RT, followed by centrifugation (250 *g* for 2 minutes), removal of supernatant, and resuspension in 2% low melting point agarose (A4018-50G, Sigma-Aldrich, United Kingdom) in 0.1 M PB. The agarose block containing the cells was then cut into sections approximately 1 mm × 1 mm × 200 μm using a razor blade. Sections were washed (4 × 15 minutes in 0.1 M PB), post-fixed in 1% reduced osmium (1% osmium tetroxide/1.5% potassium ferricyanide) for 60 minutes at 4°C, washed again (3 × 5 minutes in 0.1 M PB) and incubated in 1% tannic acid in 0.05 M PB for 45 minutes at RT. The reaction was quenched in 1% sodium sulfate in 0.05 M PB for 5 minutes at RT. After washing (3 × 5 minutes in distilled water), sections were dehydrated using a graded series of ethanol (70% × 2, 90% × 2, 100% × 3, 10 minutes each) followed by infiltration with TAAB Epon 812 resin (T004, TAAB) (75:25 ethanol: Epon for 2 hours, 50:50 ethanol: Epon for 2 hours, 25:75 ethanol: Epon for 2 hours and 100% Epon overnight). Sections were then flat embedded using Aclar (L4458, Agar Scientific, Stansted, United Kingdom) and polymerized at 60°C for 48 hours.

Ultrathin sections were cut from blocks using a 3-mm ultra 45° diamond knife (DiATOME) on an ultramicrotome (EM UC7, Leica Microsystems Milton Keynes, United Kingdom) and collected onto formvar coated slot grids, then post stained with lead citrate for 10 minutes. Sections were viewed using a transmission electron microscope (Tecnai G2 Spirit BioTwin, Thermo Fisher Scientific) at 120 kV, and images were captured using a CCD (Orius, Gatan, Pleasanton, CA).

### Metabolic tagging of parasites in the presence or absence of IMP-1002

For YnMyr (also known as YnC12 or Alk-14; tetradec-13-ynoic acid) tagging experiments, purified parasites were labeled metabolically using 25 μM YnMyr added to the culture medium. For YnPal (also known as YnC14 or Alk-16; heptadec-17-ynoic acid) labeling, the compound was stabilized by base treatment and absorbed to BSA to maximize its uptake and incorporation [39]. The required amount (for example, 120 μl of a 50 mM stock of YnPal for 240 ml RPMI 1640) was combined with 600 μl of 0.01 M NaOH and warmed to 70°C for 3 to 4 minutes, then 1.5 ml of warm 5% BSA solution was added and the mix maintained at 37°C for 3 to 4 minutes. The solution was added to the RPMI 1640 culture medium, filtered through a 0.2-μm filter, and then the parasites were fed with the YnCPal-containing medium. In all experiments, the final DMSO percentage did not exceed 0.05%.

### Preparation of *P*. *falciparum* proteins and copper(I)-catalyzed alkyne-azide cycloaddition (CuAAC) labeling

Parasites of the appropriate stage were either purified through Percoll, washed, and pelleted or directly pelleted without purification. The cell pellet was lysed in 0.15% saponin, using one and a half times the pellet volume for 10 minutes on ice. Following centrifugation, the pellet was washed further with PBS until the supernatant was free of hemoglobin and stored at −80°C until use. The pellet was thawed in 10 times its volume of 1% (v/v) Triton X-100, 0.1% (w/v) SDS in PBS containing protease inhibitors but without EDTA, sonicated for 1 minute and then left on ice for 20 minutes. Insoluble material was removed by centrifugation, and the supernatant was snap frozen and stored at −80°C. The protein concentration of the lysate was measured using a Pierce BCA Protein Assay Kit (23225, Thermo Fisher Scientific) following the manufacturer’s instructions.

The lysate was adjusted to 1 mg/mL protein with PBS, and premixed click reagents [100 μM azido-TAMRA-biotin (AzTB) capture reagent, 1 mM CuSO_4_, 1 mM Tris(2-carboxyethyl)phosphine (TCEP), 100 μM Tris[(1-benzyl-1H-1,2,3-triazol-4-yl)methyl]amine (TBTA); mixed in the order stated and pre-incubated for 2 minutes] were added at the equivalent of 6 μl click reaction mix to 100-μl protein solution [3,40]. The sample was vortexed for 1 hour at RT and the reaction quenched by the addition of 10 mM EDTA. Protein was precipitated with 2 volumes of methanol, 0.5 volumes of chloroform, and 1 volume of water. After centrifugation for 10 minutes at 17,000 *g*, the top methanol/water layer was removed, and 0.5-ml ice-cold methanol was added prior to vortexing and sonication to break up and disperse the protein disk. The protein was collected by centrifugation (17,000 *g* for 10 minutes at 4°C) and air-dried for approximately 15 minutes, then redissolved to 10 to 20 mg/ml in PBS containing 2% SDS, 10 mM DTT, with vortexing for 15 to 30 minutes.

### Protein enrichment for immunoblot analysis

For analysis of affinity purified proteins by SDS PAGE, precipitated samples were enriched using 25 μl of Neutravidin Agarose Resin for lysate containing 150 μg of protein. The resin was pre-washed 3× with 0.2% SDS in PBS followed by an enrichment of the labeled proteins from the lysate. Following the pull down for 2 hours at RT with shaking, the supernatant was removed, and the beads were washed 3 times with 0.2% SDS in PBS. Proteins were eluted by treatment of the beads with SDS-PAGE sample loading buffer containing DTT (at a final 100 mM concentration) and boiling for 10 minutes. Following a centrifugation step to remove any insoluble material, supernatants were loaded on the gel.

### Affinity purification of labeled proteins and proteomic sample preparation

After click chemistry, precipitation, and dissolution in 2% SDS in PBS, samples were diluted with PBS to 1 mg/ml protein. For proteomic analysis, labeled proteins were first enriched. An agarose mixture comprised of one-third Neutravidin Agarose resin and two-thirds Pierce Control Agarose resin (Thermo Fisher Scientific) was prepared to minimize contamination of samples with neutravidin from the beads, and 30 μl of this resin mixture was used to enrich labeled protein from lysate containing up to 300-μg protein. The resin mixture was pre-washed 3 times with 0.2% SDS in PBS using at least 5 times the bead volume, then the protein solution was incubated with the resin for 2 hours at RT, with shaking. The resin was washed sequentially 3 times with 5 to 10 volumes of 1% SDS in PBS, twice with 50 mM ammonium bicarbonate (AMBIC) containing 4 M urea, and a further 3 times with 50 mM AMBIC followed by sample processing as described previously [41]. To improve detection of cysteine-containing peptides, thiols were reduced and alkylated; proteins were reduced with 10 mM DTT in 50 mM AMBIC for 30 minutes at 55°C and alkylated with 10 mM iodoacetamide (IAA) in 50 mM AMBIC for 30 minutes at RT in the dark. Proteins were digested with trypsin overnight (0.12 μg Trypsin Gold [Promega, Southampton, United Kingdom, Cat. # V5280] for 300 μg protein). Moreover, 1.5% (v/v) trifluoroacetic acid (TFA; Thermo Scientific, United Kingdom Cat. #28902) was added to inactivate the trypsin and peptides were desalted using stop-and-go extraction (STAGE) tips and reverse phase C18 poly(styrenedivinylbenzene) polymer cation exchange (SDB-XC) membranes. The peptides were eluted in 79% acetonitrile (MeCN)/21% water and dried using a Speed Vac concentrator. Prior to liquid chromatography–tandem mass spectrometry (LC–MS/MS) analysis, samples were dissolved in 15 μl of 0.5% TFA, 2% MeCN in water using vortex, brief sonication, and a final centrifugation step at 17,000 *g* for 10 minutes at 15°C to remove insoluble material. Moreover, 11 μl of each sample was transferred to an autosampler compatible vial.

### Global proteome analysis: TMT labeling of peptides and high pH reverse fractionation

Fifty microliters of lysate from parasites grown with or without IMP-1002 were treated with methanol:chloroform:water. Precipitated protein was washed with 200 μl methanol, collected by centrifugation (17,000 *g* for 10 minutes at 4°C) and solubilized in 20 μl 50 mM TEAB containing 0.2% ProteaseMAX Surfactant (Promega UK, Cat. # V2071) for 1 to 2 hours with vortex and occasional sonication. The samples were reduced with 5 mM DTT in 50 mM TEAB for 20 minutes at 56°C and alkylated with 14.85 mM IAA in 50 mM TEAB for 30 minutes at RT in the dark. Samples were trypsin-treated (1.8 μg Trypsin Gold for 50 μg protein) in 0.05% ProteaseMAX and then TFA was added to a final concentration of 0.5%, and incubated for 5 minutes at RT, to inactivate the trypsin. Peptides were purified by STAGE-tip and reverse phase C18 SDB-XC membrane, with elution in 70% MeCN and 30% water, and dried.

The TMT10plex Label Reagent Set (Thermo Fisher Scientific, Cat # 90309) was used according to the manufacturer’s instructions. Immediately before use, the reagents were equilibrated to RT and dissolved in anhydrous MeCN. Peptides were dissolved in 25 μl 50 mM TEAB with sonication for 10 minutes, and then 0.2 mg TMT label reagent was added and each sample incubated for 1 hour. To quench the reaction, 8 μl 5% hydroxylamine were added to the sample and incubated for 15 minutes. A small quantity (about 5%) of each sample was used to check the labeling through an initial liquid chromatography–mass spectrometry (LC–MS) analysis to determine the ratio of labeled reporter ions. Prior to mixing, the ratio was corrected for any differences in labeling efficiency. Samples were combined into one tube in equal amounts and peptides were initially separated by high pH reverse fractionation with a gradient step wise elution from 5% to 50% MeCN to increase the proteome coverage, using the Thermo Fisher Scientific kit (Cat # 84868) according to the manufacturer’s instructions. Each fraction was then dried and redissolved in 15 μl 0.1% TFA to allow 10 μl per injection.

### Proteomic data acquisition and analysis

For the peptides from proteins labeled with YnMyr in the presence or absence of IMP-1002, data were acquired on a Q-Exactive Hybrid Quadrupole-Orbitrap mass spectrometer (Thermo Scientific) with a 120-minute acquisition time. Peptides were resolved chromatographically on an Ultimate 3000 RS-LC nano system (Thermo Scientific) using a 50 cm × 75 μm EASY-Spray C18 column (Thermo Scientific) at a flow rate of 250 nl/min. The elution conditions comprised a gradient of solutions A (0.1% aqueous formic acid [FA] in water) and B (0.1% FA in MeCN) over 2 hours. Via nano electrospray ionization, the eluent was introduced to the Q Exactive, which was operated in data-dependent mode using a survey scan of 350 to 1,650 m/z at a resolution of 70,000. Up to 10 of the most abundant isotope patterns with 2+ charge or higher from the survey scan were selected with an isolation window of 2.0 m/z and fragmented by higher-energy C-trap dissociation (HCD) with normalized collision energies of 25%. Subsequent scans were acquired at a resolution of 17,500 from m/z 200 to 2,000.

For the whole proteome, analysis was performed on an Orbitrap Fusion Lumos Tribrid mass spectrometer (Thermo Scientific) with a 120-minute acquisition time. Peptides were resolved chromatographically on an Ultimate 3000 RS-LC-nano System (Thermo Scientific), using a 50 cm × 75 μm EASY-Spray C18 column (Thermo Scientific) at a flow rate of 300 nl/min. The elution conditions comprised a gradient starting at 2% B (0.1% FA, 80% MeCN and water) and 98% A (0.1% FA in water) and increasing to 27.5% B over 110 minutes followed by an increase to 40% B over 10 minutes and a final increase to 90% B over 1 minute. Via nano electrospray ionization, the eluent was introduced into the Orbitrap Fusion Lumos, which was operated in “TMT acquisition mode,” and peptides were analyzed using a 375 to 1,500 m/z scan range using quadrupole isolation at 120,000 resolution for an ion at 200 m/z. Tandem mass spectra were first collected using the ion trap and fragmented using 35% collisional induced dissociation (CID). A dynamic exclusion list was employed to prevent repeat sampling (repeat count of 2, repeat duration of 15 seconds, exclusion list size 100, and exclusion duration of 30 seconds).

### Proteome data analysis

Peptides identification and quantification were conducted using MaxQuant software (versions 1.5.3.8 for YnMyr labeling and label free quantitation, and 1.6.0.13 for the whole proteome with TMT quantitation) using the PlasmoDB-29_Plasmodium3D7_Annotated Protein database. All mass spectrometry “.raw” files were loaded directly into the MaxQuant software. Protein intensity values were calculated based on the intensities of their corresponding peptides, and analyses of both LFQ (YnMyr labeling) and TMT (whole proteome) experiments in MaxQuant were performed using the built-in algorithms. Cysteine carbamidomethylation was selected as a fixed modification, and methionine oxidation and N-terminal acetylation as variable modifications. For the YnMyr labeling and purification experiment, myristoylation was set as a variable modification using a composition of C(22) H(37) N(7) O(4) with a monoisotopic mass of 463.2907 on any N-terminus. For enzyme digestion, trypsin was selected, which allows cleavage carboxyl terminus of Arg and Lys residues and LysC, which allows cleavage after Lys residues. Up to 2 missed cleavages were allowed. The FDR was set to 0.01 for peptides, proteins and sites. Other parameters were used as preset in the software. “Unique and razor peptides” mode was selected to allow identification and quantification of proteins in groups (razor peptides are uniquely assigned to protein groups and not to individual proteins), and all identifications were based on at least 2 unique peptides. The data were analyzed using Perseus version 1.5.6.0, Microsoft Excel 2010, and GraphPad Prism version 8 for all experiments.

MS data were also processed with PEAKS X+, which as a default performs de novo peptide sequencing prior to database searches, in order to improve the accuracy of the results. The software also searches for common PTMs (PEAKS PTM) and point mutations (SPIDER). The data were searched against the same database used in MaxQuant analyses. Trypsin was selected for database searches. The maximal mass error was set to 5 ppm for precursor ions and 0.01 Da for product ions. Cysteine carbamidomethylation was set as fixed modification and methionine oxidation and myristoylation (463.2907 on any N-terminus) were set as variable modifications. The maximal number of modifications per peptide was set as 3. The FDR was set to 0.01 for peptides, and a minimum of 1 unique peptide per protein was required.

### Generation of repair and Cas-9 plasmids for the G2A/G2G competition screen of ARO, CDPK1, GAP45, ISP3, S9C, and TRP

For each locus, a rescue plasmid was used with 200 base pair homology regions either side of the G2A mutation and guide sequence. The sequence between the G2A mutation and the guides was recodonized using the Codon Usage Table from PlasmoDB [42]. The tool on the ctegd.uga.edu/ website was used to determine 2 guides for each target gene based on close proximity to the G2A mutation, total score as calculated by use of an efficiency score [43] and the CRISPRRater [44]. Each construct was flanked by unique restriction sites (SacI and SacII) not present in any of the constructs or the pMK-RQ kanamycin resistance plasmid from GeneArt, for linearization prior to transfection. For each construct a second plasmid contained the same homology arms and retained a codon for glycine at position 2 with a synonymous mutation from the endogenous sequence.

### Analysis of the G2A screen by DNA sequencing

The G2A screen was analyzed by Illumina MiSeq. Parasite genomic DNA was extracted and specific integration-selective primers containing a MiSeq adapter sequence were used to amplify a 289 to 466 bp fragment (depending on construct) around the codon encoding G2A/G2G. The PCR was performed, and samples were cleaned as recommended by the manufacturer for preparation of the 16S Metagenomic sequencing library (Part # 15044223). To increase the number of sequence reads per sample, the KAPA HyperPrep kit was used according to the manufacturer’s instructions to label the PCR fragments by ligating indices at each end creating a unique barcode for each sample. A 9 bp sequence around the glycine or alanine codon was used to determine the ratio of integrated G2A versus G2G.

### Cloning of constructs and transfection of *P*. *falciparum*

The *gap45*:*ha3*:*loxP*::*comp_gap45[G2A]* construct was generated through PCR, digest, ligation, and cloning using the *gap45*:*ha3*:*loxP*::*comp_gap45* construct and the same guide [19] and cloned into the pDC2-cam-Cas-9-U6-hDHFRyFCU-plasmid [36,45,46]. Guide and rescue plasmids were paired and ethanol precipitated prior to transfection. *P*. *falciparum gap45*:*ha3*:*loxP* (B11 background) [19] or 3D7 parasites were used. For transfection, mature schizonts were electroporated using the Amaxa 4D electroporator (Lonza, Slough, United Kingdom) and the P3 Primary cell 4D Nucleofector X Kit L (Lonza) and program FP158 [47], with 60 μg of linearized rescue plasmid and 20 μg of the CRISPR/Cas-9 plasmid carrying the respective guide RNA. Selections were carried out as recently described [36]; parasites were cultured in the presence of 2.5 nM WR99210 for 5 days to select for parasites with the Cas-9/guide plasmid. Transfected parasites were detected after 22 days, and DNA integration was confirmed by PCR amplification. Parasites were then treated with 1 μM 5-fluorocytosine (Ancotil) to remove residual Cas-9/guide plasmid and cloned by limiting dilution after 37 days [48]. Individual clones were then screened by PCR amplification to confirm integration of the required DNA sequence.

### Analysis of parasite growth and invasion

To analyze the growth of *gap45*:*ha3*:*loxP*::*comp_gap45[G2A]* Clone 01 and Clone 02 as well as *gap45*:*ha3*:*loxP* and *gap45*:*ha3*:*loxP*::*comp_gap45[WT]*, parasites were adjusted to a parasitemia of 0.1% and treated with rapamycin or DMSO. At the beginning of the assay (in cycle 0) and at 72 hours (cycle 1) and 120 hours (cycle 2) PI, when parasites were at a late ring/early trophozoite stage, samples were processed and analyzed by flow cytometry. Each experiment was set up in triplicate, and these biological replicates were complemented with the use of the 2 clones, which served as an additional biological repeat.

The invasive capacity of genetically modified parasites (*gap45*:*ha3*:*loxP* / *gap45*:*ha3*:*loxP*::*comp_gap45[WT]* and *gap45*:*ha3*:*loxP*::*comp_gap45[G2A]* treated with either DMSO or rapamycin), and 3D7 parasites treated with either 140 nM IMP-1002 or DMSO, was measured using Percoll-purified synchronized mature schizonts added to RBC at 1% hematocrit and a parasitemia of 1% to 3%. Samples were fixed with 4% PFA and 0.02% glutaraldehyde at 0, 4, and 24 hours later, enabling the percentage of newly formed ring-infected RBCs to be determined by Hoechst staining and flow cytometry. Experiments were performed in triplicate with blood from 3 different donors. The data were analyzed with FlowJo and GraphPad Prism software to determine the standard deviation and perform a *t* test for statistical significance of differences between the samples.

### APE analysis of protein thioesters

For APE analysis, a parasite lysate (in 1% Triton X-100, 0.1% SDS, and EDTA-free protease inhibitor cocktail) was treated as described previously [23]. Each lysate was adjusted to 2 mg/ml protein, and 92.5 μl of samples per condition were treated to reduce Cys residues with 5 μl neutralized TCEP at a final concentration of 10 mM for 30 minutes with nutation. These free Cys residues were then blocked by alkylation with 2.5 μl of NEM (freshly prepared 1 M solution, diluted to 25 mM final concentration). The reaction was stopped by protein precipitation using methanol–chloroform–water (4:1.5:3) with sequential addition of 400-μl methanol, 150-μl chloroform, and 300-μl water (all pre-chilled on ice). Following centrifugation (20,000 *g* for 5 minutes at 4°C), the methanol/aqueous layer was removed, 1-ml pre-chilled methanol was added, and after mixing, the protein was pelleted by centrifugation at 20,000 *g* for 3 minutes at 4°C. The pellet was washed again with pre-chilled methanol and dried under vacuum (Centrivap Concentrator, Labconco, Kansas City, MO). To ensure complete removal of NEM from the protein pellet, each sample was resuspended in 100 μl 50 mM triethanolamine, pH 7.3, 150 mM NaCl containing 1× protease inhibitor mixture (Roche, Welwyn Garden City, United Kingdom), 5 mM PMSF (Sigma, United Kingdom), 5 mM EDTA (Fischer, United Kingdom), and 1,500 units/mL benzonase (TEA buffer) [23], containing 4% SDS, warmed to 37°C for 10 minutes, and briefly (approximately 5 seconds) sonicated (Ultrasonic Cleaner, VWR, Lutterworth, United Kingdom), with 2 additional rounds of methanol–chloroform–water precipitation.

For hydroxylamine (NH_2_OH) cleavage of palmitoyl thioester bonds and subsequent alkylation of the cysteines with mPEG-Mal, the protein pellet was redissolved in 100 μl TEA buffer containing 4% SDS, 4 mM EDTA, and split into two 50-μl samples. One sample was treated with 150 μl TEA buffer pH 7.3, containing 0.2% Triton X-100 and 3 M NH_2_OH at a final concentration of 0.75 M NH_2_OH. The second control sample was not treated with NH_2_OH but diluted with 150 μl of TEA buffer, 0.2% Triton X-100. After incubation at RT for 1 hour with nutation, the protein was precipitated with methanol–chloroform–water and redissolved in 100-μl TEA containing 4% SDS, 4 mM EDTA, warmed to 37°C for 10 minutes, and briefly (approximately 5 seconds) sonicated. Next, to each sample was added 150 μl TEA buffer containing 0.2% Triton X-100 and 4 mM mPEG-Mal (10 kDa; Sigma) for a final concentration of 1 mM mPEG-Mal. Samples were incubated for 2 hours at RT with nutation before a final methanol–chloroform–water precipitation. The protein precipitate was redissolved as described above, and samples containing 10 μg protein were resolved by 3% to 12% gradient Bis-Tris SDS-PAGE and analyzed by western blot using rabbit anti-GAP45 and anti-CDPK1 antibodies.

## Supporting information

S1 Table Guide RNA sequences. (PDF)

S2 Table Oligonucleotides used for PCR and sequence analysis. (PDF)

S3 Table Percentage of PEGylated and non-PEGylated protein in cell extracts measured by western blotting with anti-GAP45 and anti-CDPK1 antibodies (see Fig 7C). CDPK1, calcium-dependent protein kinase 1; GAP45, glideosome-associated protein 45.
(PDF)

S1 Fig Inhibition of NMT during schizogony leads to a block in parasite development. Parasitemia was significantly reduced in IMP-1002–treated culture compared with DMSO-treated control. Parasites were treated with IMP-1002 or DMSO from 34 to 45 hours PI. At the first sign of egress in the DMSO control (at 45 hours PI), the growth medium was exchanged to drug-free medium, and the parasites were quantified by flow cytometry 6 (51 hours PI) and 31 (76 hours PI) hours later. Data are from 3 technical replicates (see S1 Data). The difference in parasitemia was significant (*p* < 0.0001 [****] for both 51 hours PI and 76 hours PI, but not at 45 hours PI [ns.]; unpaired Student *t* test with Welch correction not assuming an equal SD for each time point individually. NMT, *N*-myristoyl transferase.
(PDF)

S2 Fig IMP-1002 treatment during schizogony changes the differential solubility of ARO and CDPK1. To examine the differential solubility of proteins present in IMP-1002–treated and untreated parasites, Percoll-purified schizonts were lysed and sequentially fractionated using hypotonic and high salt buffers (to solubilize cytoplasmic proteins), sodium carbonate (to solubilize peripheral membrane proteins), and a buffer containing 1% Triton X100 and 0.1% SDS (to solubilize membrane proteins). These fractions together with the insoluble pellet, were analyzed by western blot using antibodies to ARO, CDPK1, GAP45, HSP70, MSP7, and MTIP. In the presence or absence of IMP-1002, ARO was largely in the hypotonic soluble and carbonate insoluble fractions, respectively; CDPK1 was distributed in the hypotonic/high salt soluble and carbonate insoluble fractions, respectively, under the same conditions. The mobility of molecular mass markers is indicated on the right side for each protein. ARO, armadillo domain–containing rhoptry protein; CDPK1, calcium-dependent protein kinase 1; GAP45, glideosome-associated protein 45; HSP70, heat shock protein 70; MSP7, merozoite surface protein 7; MTIP, myosin tail interacting protein.
(PDF)

S3 Fig Direct identification of the modified N-terminal glycine of 2 NMT substrates by mass spectroscopy. Modified N-terminal peptides from A. Metal-dependent protein phosphatase 6 (PF3D7_1309200) and B. Putative acylated pleckstrin-homology domain containing protein (APH). The N-terminal modification and the peptide sequence is deduced from the parent ion mass and fragmentation pattern. The b1 ion (521.32, which correspond to the N-terminal glycine modified with YnMyr) is diagnostic of the metabolic incorporation of YnMyr by NMT into the protein. NMT, *N*-myristoyl transferase.
(PDF)

S4 Fig A G2G/G2A CRISPR/Cas-9-mediated viability screen. **(A)** For each gene of interest (ARO, CDPK1, GAP45, ISP3, S9C, and TRP) 2 guide sequences were selected, except for *aro* for which the guide design was limited to one because the first exon was too short. **(B)** Two repair plasmids with either a G2A point mutation or a silent G2G mutation were generated. **(C)** The plasmids were mixed at a 50:50 ratio before linearization in the sequence flanking both homology arms. **(D)** Integration was facilitated by CRISPR/Cas-9, and then following successful transfection, parasite genomic DNA was extracted. **(E)** Integration specific primers allowed a selective PCR amplification of the integrated fragment, to which MiSeq adapter sequences were attached. **(F)** The Illumina adapters were attached by ligation using the KAPA HyperPrep Kit. **(G)** To discriminate between samples, the adapter-ligated fragments were labeled with indices by indexing PCR with 2 indices at each end creating a unique barcode. This step also added the attachment site for the MiSeq instrument (P5 or P7). **(H)** Following Illumina sequencing, the ratio of the number of sequences for either the G2A or G2G variant provided an indication of the viability of each variant. The number of total reads with either the G2A or G2G sequence, for either 2 or 3 experiments, is indicated (see also S1 Data for the distribution of reads). ARO, armadillo domain–containing rhoptry protein; CDPK1, calcium-dependent protein kinase 1; GAP45, glideosome-associated protein 45; TRP, tetratricopeptide repeat protein.
(PDF)

S5 Fig Construction of a GAP45 gene complementation parasite line. **(A)** The genetic complementation strategy used to introduce a second copy of the *gap45* gene coding for either an N-terminal glycine (G2G) or alanine (G2A) and its promoter sequence into the *pfs47* locus of a *gap45*:*ha3*:*loxP* parasite clone in the DiCre-expressing *P*. *falciparum* line B11, generating the *gap45*:*ha3*:*loxP*::*comp_gap45[WT]* (cGAP45[WT]) and *gap45*:*ha3*:*loxP*::*comp_gap45[G2A]* (cGAP45[G2A]) lines. **(B)** Schematic representation of the Pf47 locus with and without integration of the *gap45* gene, and the oligonucleotide primers within the 5′ and 3′ UTR regions used to analyze integration by PCR. **(C)** PCR analysis of *gap45*:*ha3*:*loxP*::*comp_gap45[WT]* and *gap45*:*ha3*:*loxP*::*comp_gap45[G2A]* uncloned parasites showing 5′ and 3′ UTR integration. The B11 (3D7) line was used as a control for the WT parasite. **(D)** PCR analysis of 3 selected *gap45*:*ha3*:*loxP*::*comp_gap45[G2A]* clones. The B11 3D7 line was used as a control for the WT parasite. GAP45, glideosome-associated protein 45; WT, wild-type.
(PDF)

S6 Fig Analysis of gene excision by PCR in *gap45*:*ha3*:*loxP*, *gap45*:*ha3*:*loxP*::*comp_gap45[WT]*, and *gap45*:*ha3*:*loxP*::*comp_gap45[G2A]* parasite clones. **(A)** Schematic representation of the complementation locus, the gap45 locus and the gap45 locus after rapamycin induced diCre-mediated excision, indicating the oligonucleotides used to analyze the parasites treated with rapamycin or DMSO by PCR, and oligonucleotides used to check presence of complemented construct after rapamycin treatment. **(B)** Rapamycin induces excision at the *gap45* locus in all 4 parasite lines. **(C)** Rapamycin has no effect on the complementation locus containing either the *gap45[WT] or gap45[G2A]* genes. GAP45, glideosome-associated protein 45; WT, wild-type.
(PDF)

S1 Data Data underlying the summary figures for parasite growth. The data for experiments summarized in the figures are included in a single Excel file as individual worksheets for Figs 1A, 1B, 4, 5C, 8B and 8C and S1 Fig.
(XLSX)

S2 Data All proteins from parasites metabolically labeled with YnMyr in presence or absence of IMP-1002 and bound and eluted from the Neutravidin column. These data underpin Fig 3A.
(XLSX)

S3 Data All proteins from parasites treated with either IMP-1002 or DMSO. These quantitative proteome data underpin Fig 3B.
(XLSX)

S4 Data Proteins significantly reduced in abundance following treatment with IMP-1002. These data are the basis of the summary provided as Fig 3C.
(XLSX)

S1 Raw Images The original images used to compile Figs 5A, 6B and 7A–7C and S2, S5C, S5D, S6B and S6C Figs are included in this file, and each image is annotated to indicate the relevant figure in the manuscript. (PDF)

## Abbreviations

2-BP: 2-bromopalmitate
AMBIC: ammonium bicarbonate
APE: acyl-PEG exchange
ARO: armadillo domain–containing rhoptry protein
BSA: bovine serum albumin
CDPK1: calcium-dependent protein kinase 1
CID: collisional induced dissociation
ER: endoplasmic reticulum
FA: formic acid
FDR: false discovery rate
GAP45: glideosome-associated protein 45
GO: gene ontology
GPI: glycosyl phosphatidylinositol
HCD: higher-energy C-trap dissociation
HSP70: heat shock protein 70
IAA: iodoacetamide
IFA: immunofluorescence assay
IMC: inner membrane complex
ISP3: IMC sub-compartment protein 3
LC–MS/MS: liquid chromatography–tandem mass spectrometry
LFQ: label-free quantification
MFI: median fluorescence intensity
mPEG-Mal: methoxy(polyethylene glycol)-maleimide
MTIP: myosin tail interacting protein
MyoA: myosin A
NAT: N-α-acetylation
NEM: N-ethylmaleimide
NMT: *N*-myristoyl transferase
NMTi: NMT inhibitor
NPM: N-terminal protein modification
PFA: paraformaldehyde
PI: postinvasion
PM: plasma membrane
RBC: red blood cell
RBP: ribosome-associated protein biogenesis factor
RON4: rhoptry neck protein 4
RT: room temperature
STAGE: stop-and-go extraction
TBTA: Tris[(1-benzyl-1H-1,2,3-triazol-4-yl)methyl]amine
TCEP: Tris(2-carboxyethyl)phosphine
TFA: trifluoroacetic acid
TMT: tandem mass tag
TRP: tetratricopeptide repeat protein
WT: wild-type
