# Supplementary material for: Inhibition of protein N-myristoylation blocks Plasmodium falciparum intraerythrocytic development, egress and invasion
Source: PLoS Biol. 2021 Oct 25;19(10):e3001408. doi: 10.1371/journal.pbio.3001408 (PMC8544853; doi:10.1371/journal.pbio.3001408)
Supplement: S1 Table — (PDF) [file pbio.3001408.s001.pdf]

S1 Table. Guide RNA sequences

Guide name	Sequence
ARO_G2A_Guide-01_F	ATTGATGGGAAATAATTGCTGTGC
ARO_G2A_Guide-01_R	AAACGCACAGCAATTATTTCCCAT
ARO_G2A_Guide-02_F	ATTGCTTCTGCTAAGTCTTGCTCA
ARO_G2A_Guide-02_R	AAACTGAGCAAGACTTAGCAGAAG
CDPK1_Guide_01_F	ATTGAGGAGAAGTAAATTTACGAA
CDPK1_Guide_01_R	AAACTTCGTAAATTTACTTCTCCT
CDPK1_Guide_02_F	ATTGTTACGAATGGAAATAATTAT
CDPK1_Guide_02_R	AAACATAATTATTTCCATTCGTAA
GAP45_Guide_02_F	ATTGATGTTCAAGAAGCAAAGTAA
GAP45_Guide_02_R	AAACTTACTTTGCTTCTTGAACAT
GAP45_Guide_03_R	AAACAACGTAAAGATATTGATGAA
GAP45_Guide_03_F	ATTGTTCATCAATATCTTTACGTT
ISP3_Guide_01_F	ATTGTTTCAGGACAATGAGATATA
ISP3_Guide_01_R	AAACTATATCTCATTGTCCTGAAA
ISP3_Guide_02_F	ATTGTAAGAGTGGAAATAGTATAA
ISP3_Guide_02_R	AAACTTATACTATTTCCACTCTTA
S9C_Guide_01_R	AAACACTAATCTTCAGACCGCACC
S9C_Guide_01_F	ATTGGGTGCGGTCTGAAGATTAGT
S9C_Guide_02_R	AAACCACCCACCTAGCTATTCAAA
S9C_Guide_02_F	ATTGTTTGAATAGCTAGGTGGGTG
TRP_Guide_01_F	ATTGTGCATTCGGAAGTAAGAATT
TRP_Guide_01_R	AAACAATTCTTACTTCCGAATGCA
TRP_Guide_02_F	ATTGTTATGCTAGCATGAAATTGT
TRP_Guide_02_R	AAACACAATTTCATGCTAGCATAA
