# Supplementary material for: Inhibition of protein N-myristoylation blocks Plasmodium falciparum intraerythrocytic development, egress and invasion
Source: PLoS Biol. 2021 Oct 25;19(10):e3001408. doi: 10.1371/journal.pbio.3001408 (PMC8544853; doi:10.1371/journal.pbio.3001408)
Supplement: S2 Table — (PDF) [file pbio.3001408.s002.pdf]

S2 Table. Oligonucleotides used for PCR and sequence analysis

Name	Sequence
ARO_Intg_F1	TATTATACCGTTGCCTTTCAAATGGCGGG
ARO_Intg_R1	CATACATCTCTACCGGCGCAACAG
GAP45_Intg_F1	aaacgtatggaagtgtaaaggg
GAP45_Intg_R1	CTCGTCTATGTCCTTTCTCTTTGGC
ISP3_Intg_F1	GGAAAAAGTTTTATGTTTCAGTCTGAATATTACC
ISP3_Intg_R1	CTTTAATTGAGTTACCTGATTTGTACTTGTTAATCC
S9C_Intg_F1	gaaatataaatacatgttaacacatagttataagataatacc
S9C_Intg_R1	TTACTGTAACTTGGAGGATGTGGCC
TRP_Intg_F1	GAAAATACATTTCTTCTTAATTCATATTG
TRP_Intg_R2	CCACTTCTAATAACTTCATACTTGCG
CDPK1_Intg_F1	cattttgatggtggcacttgcctttttgagg
CDPK1_Intg_R1	ACCGTAGTTGTTACCGTTTGTGAACTTTGACC
CDPK1_WT_R1	CCATTCGTAAATTTACTTCTCCTCG
ARO_WT_R1	ATCTCTTCCTGCACAGCAATTATTTC
GAP45_WT_R1	TTCATCAATATCTTTACGTTTGGGTTCC
ISP3_WT_R1	CTGAAATATATCTATATTTGATTTACTG
S9C_WT_R1	CTTTGAATAGCTAGGTGGGTGCGGTCTG
TRP_WT_R1	TTTCATGCTAGCATAATTGTAATACTCC
ARO_MiSeq_F1	TCGTCGGCAGCGTCAGATGTGTATAAGAGACAGTATTATACCGTTGCCTTTCAAATGGC
ARO_MiSeq_R1	GTCTCGTGGGCTCGGAGATGTGTATAAGAGACAGCATACATCTCTACCGGCGCAACAG
GAP45_MiSeq_F1	TCGTCGGCAGCGTCAGATGTGTATAAGAGACAGtaaacgtatggaagtgtaaaggg
GAP45_MiSeq_R1.2	GTCTCGTGGGCTCGGAGATGTGTATAAGAGACAGCTCGTCTATGTCCTTTCTCTTTGGC
ISP3_MiSeq_F1	TCGTCGGCAGCGTCAGATGTGTATAAGAGACAGGGAAAAAGTTTTATGTTTCAGTCTGAATATTACC
ISP3_MiSeq_R1	GTCTCGTGGGCTCGGAGATGTGTATAAGAGACAGCTTTAATTGAGTTACCTGATTTGTACTTGTTAATCC
S9C_MiSeq_F1	TCGTCGGCAGCGTCAGATGTGTATAAGAGACAGgaaatataaatacatgttaacacatagttataagataatacc
S9C_MiSeq_R1	GTCTCGTGGGCTCGGAGATGTGTATAAGAGACAGTTACTGTAACTTGGAGGATGTGGCC
TRP_MiSeq_F1	TCGTCGGCAGCGTCAGATGTGTATAAGAGACAGGAAAATACATTTCTTCTTAATTCATATTG
TRP_IMiSeq_R2	GTCTCGTGGGCTCGGAGATGTGTATAAGAGACAGCCACTTCTAATAACTTCATACTTGCG
CDPK1_MiSeq_F1	TCGTCGGCAGCGTCAGATGTGTATAAGAGACAGgaaaaataaaatcgtagaaaatgtttcccc
CDPK1_MiSeq_R1	GTCTCGTGGGCTCGGAGATGTGTATAAGAGACAGGTAGTTGTTACCGTTTGTGAACTTTGACC
GAP45_MiSeq_F1.2	TCGTCGGCAGCGTCAGATGTGTATAAGAGACAGtaaacgtatggaagtgtaaaggg
AJP_161	gtgtatatttaccttacatttatctcc
AJP_162	tatgacttgttcacttgctagtgtac
AJP_169	gccaactgtagttgggtcatc
AJP_164	cattcctaacacattatgtgtataaca
AS_GAP45C_Intg_WT3UTR	gtcaggggattaaaataaaaatc
AJP_163	ATTGAGCAGAGGATATGCGCATAATGGT
G45_WTC_pbDT3_R	GCACACAACATACACATTTTTACAG
Integ_G45_3UTR_R	gatttcgatgaaattttaattttttttaatc
AJP_93	tgtttaatacatactgtgtaatcctt

Upper and lower case sequences are from exons and non-coding regions, respectively.
