# Supplementary material for: Inhibition of protein N-myristoylation blocks Plasmodium falciparum intraerythrocytic development, egress and invasion
Source: PLoS Biol. 2021 Oct 25;19(10):e3001408. doi: 10.1371/journal.pbio.3001408 (PMC8544853; doi:10.1371/journal.pbio.3001408)
Supplement: S3 Table — CDPK1, calcium-dependent protein kinase 1; GAP45, glideosome-associated protein 45. (PDF) [file pbio.3001408.s003.pdf]

S3 Table. Percentage of PEGylated and non-PEGylated protein in cell extracts measured by western blotting with anti-GAP45 and anti-CDPK1 antibodies. See Fig 7C

Protein		Parasite line, % of protein signal			
		gap45:ha3:loxP::comp_gap45[WT]		*gap45:ha3:loxP::comp_gap45[G2A]*	
	Rapamycin	-	+	-	+
**GAP45**	2-PEG-protein	13	8	13	15
	1-PEG-protein	49	44	51	43
	protein	38	48	36	41
**CDPK1**	1-PEG-protein	33	28	29	26
	protein	67	72	71	74
