# Supplementary figures and images for: Inhibition of protein N-myristoylation blocks Plasmodium falciparum intraerythrocytic development, egress and invasion
Source: PLoS Biol. 2021 Oct 25;19(10):e3001408. doi: 10.1371/journal.pbio.3001408 (PMC8544853; doi:10.1371/journal.pbio.3001408)

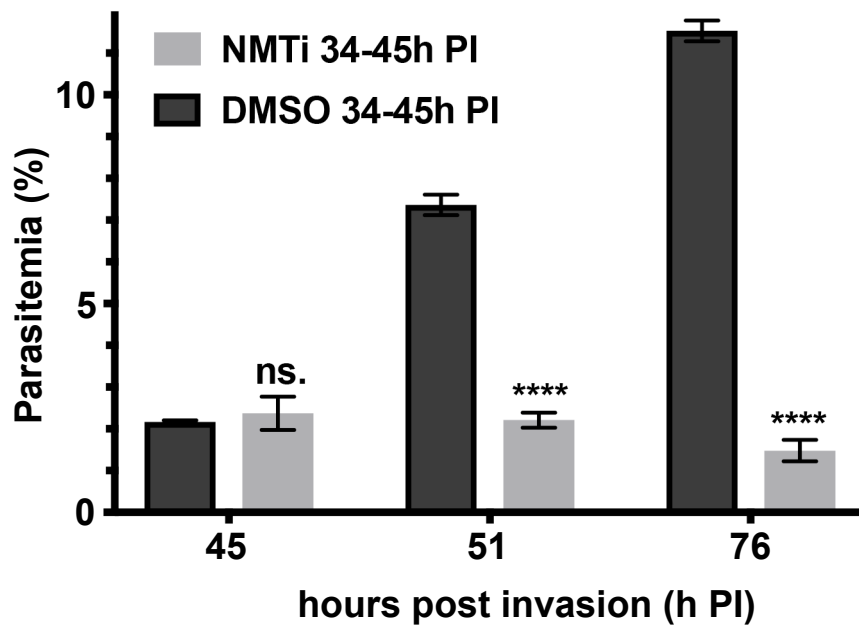

Supplement: S1 Fig — Parasitemia was significantly reduced in IMP-1002–treated culture compared with DMSO-treated control. Parasites were treated with IMP-1002 or DMSO from 34 to 45 hours PI. At the first sign of egress in the DMSO control (at 45 hours PI), the growth medium was exchanged to drug-free medium, and the parasites were quantified by flow cytometry 6 (51 hours PI) and 31 (76 hours PI) hours later. Data are from 3 technical replicates (see S1 Data). The difference in parasitemia was significant (p < 0.0001 [****] for both 51 hours PI and 76 hours PI, but not at 45 hours PI [ns.]; unpaired Student t test with Welch correction not assuming an equal SD for each time point individually. NMT, N-myristoyl transferase. (PDF) [file pbio.3001408.s004.pdf]

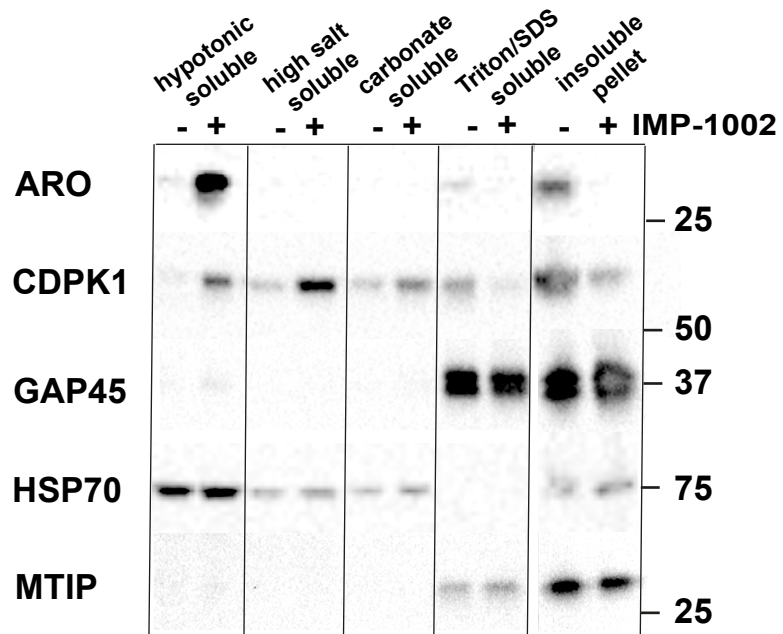

Supplement: S2 Fig — To examine the differential solubility of proteins present in IMP-1002–treated and untreated parasites, Percoll-purified schizonts were lysed and sequentially fractionated using hypotonic and high salt buffers (to solubilize cytoplasmic proteins), sodium carbonate (to solubilize peripheral membrane proteins), and a buffer containing 1% Triton X100 and 0.1% SDS (to solubilize membrane proteins). These fractions together with the insoluble pellet, were analyzed by western blot using antibodies to ARO, CDPK1, GAP45, HSP70, MSP7, and MTIP. In the presence or absence of IMP-1002, ARO was largely in the hypotonic soluble and carbonate insoluble fractions, respectively; CDPK1 was distributed in the hypotonic/high salt soluble and carbonate insoluble fractions, respectively, under the same conditions. The mobility of molecular mass markers is indicated on the right side for each protein. ARO, armadillo domain–containing rhoptry protein; CDPK1, calcium-dependent protein kinase 1; GAP45, glideosome-associated protein 45; HSP70, heat shock protein 70; MSP7, merozoite surface protein 7; MTIP, myosin tail interacting protein. (PDF) [file pbio.3001408.s005.pdf]

A. Excision PCR

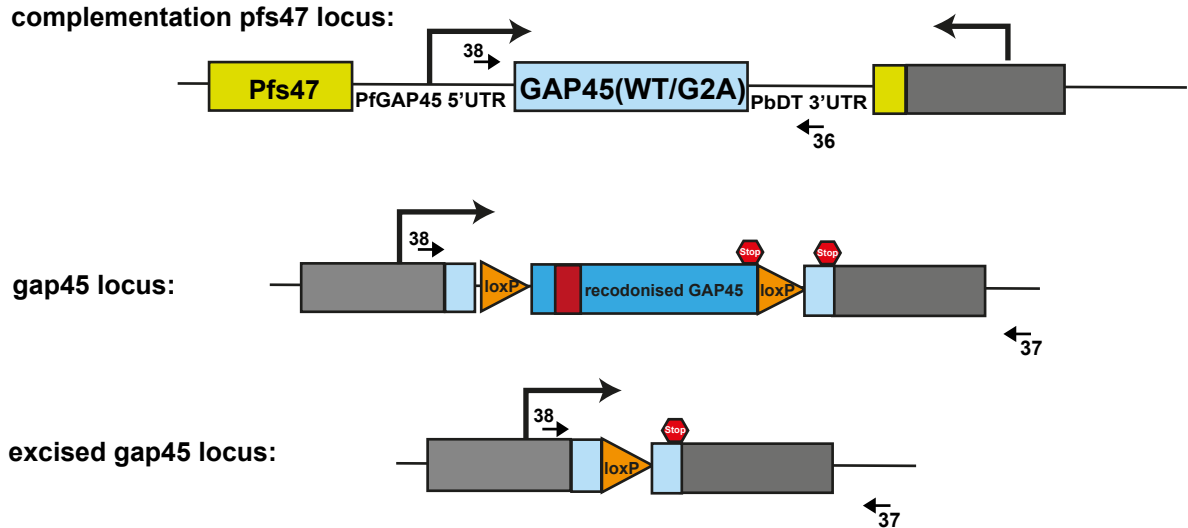

B.

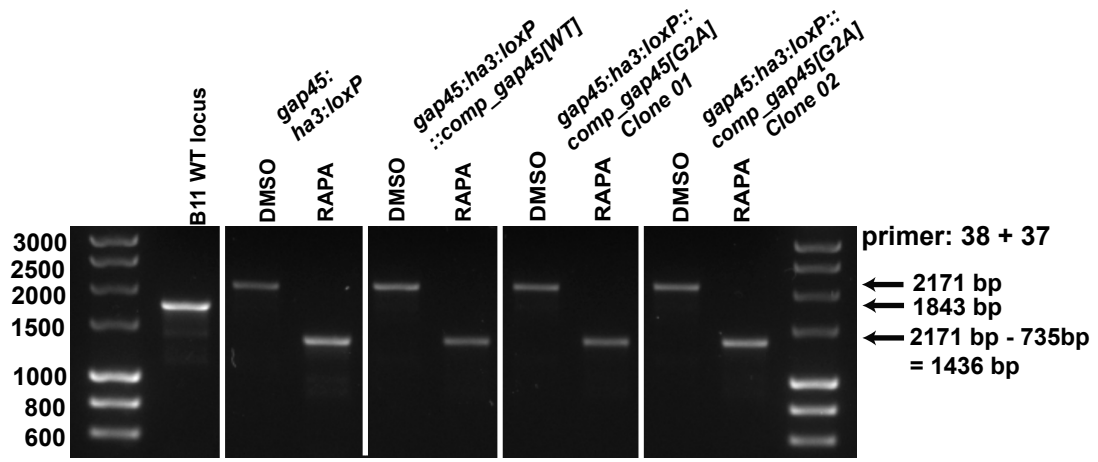

C.

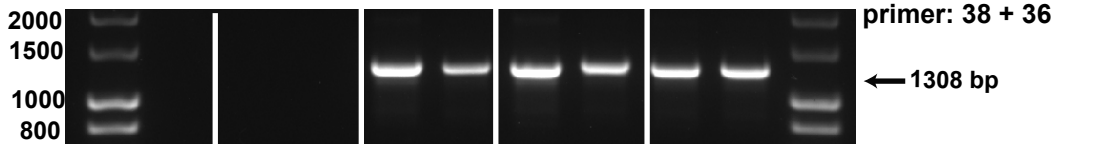

Supplement: S6 Fig — (A) Schematic representation of the complementation locus, the gap45 locus and the gap45 locus after rapamycin induced diCre-mediated excision, indicating the oligonucleotides used to analyze the parasites treated with rapamycin or DMSO by PCR, and oligonucleotides used to check presence of complemented construct after rapamycin treatment. (B) Rapamycin induces excision at the gap45 locus in all 4 parasite lines. (C) Rapamycin has no effect on the complementation locus containing either the gap45[WT] or gap45[G2A] genes. GAP45, glideosome-associated protein 45; WT, wild-type. (PDF) [file pbio.3001408.s009.pdf]
