# Supplementary material for: Inhibition of protein N-myristoylation blocks Plasmodium falciparum intraerythrocytic development, egress and invasion
Source: PLoS Biol. 2021 Oct 25;19(10):e3001408. doi: 10.1371/journal.pbio.3001408 (PMC8544853; doi:10.1371/journal.pbio.3001408)

A. Modified N-terminal peptide of metal-dependent protein phosphatase PPM6 (PF3D7_1309200).

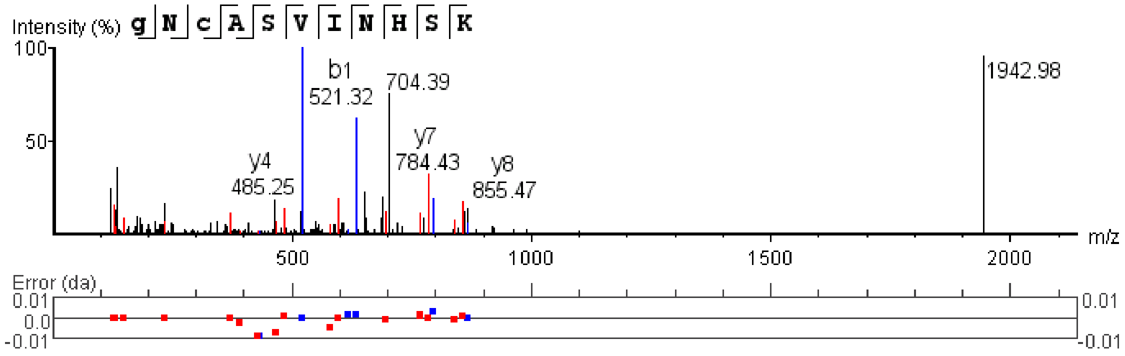

B. Modified N-terminal peptide of acylated pleckstrin-homology domain containing protein (PF3D7_0414600).

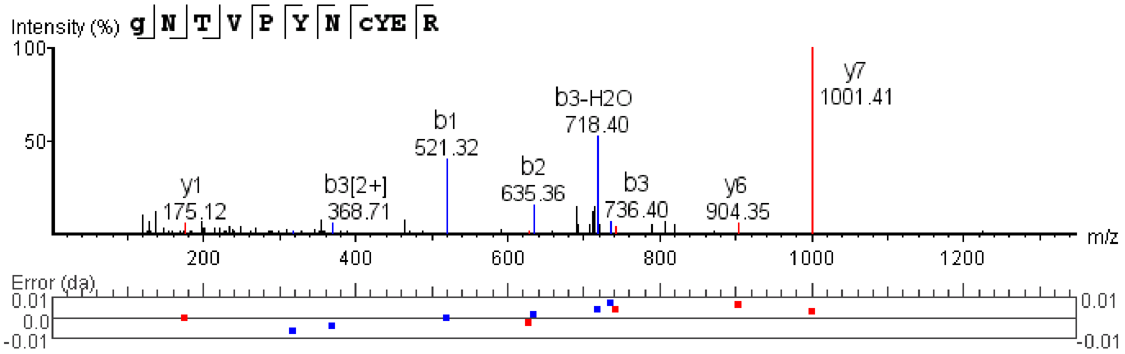

Supplement: S3 Fig — Modified N-terminal peptides from A. Metal-dependent protein phosphatase 6 (PF3D7_1309200) and B. Putative acylated pleckstrin-homology domain containing protein (APH). The N-terminal modification and the peptide sequence is deduced from the parent ion mass and fragmentation pattern. The b1 ion (521.32, which correspond to the N-terminal glycine modified with YnMyr) is diagnostic of the metabolic incorporation of YnMyr by NMT into the protein. NMT, N-myristoyl transferase. (PDF) [file pbio.3001408.s006.pdf]
