# Supplementary material for: Inhibition of protein N-myristoylation blocks Plasmodium falciparum intraerythrocytic development, egress and invasion
Source: PLoS Biol. 2021 Oct 25;19(10):e3001408. doi: 10.1371/journal.pbio.3001408 (PMC8544853; doi:10.1371/journal.pbio.3001408)
Supplement: S4 Fig — (A) For each gene of interest (ARO, CDPK1, GAP45, ISP3, S9C, and TRP) 2 guide sequences were selected, except for aro for which the guide design was limited to one because the first exon was too short. (B) Two repair plasmids with either a G2A point mutation or a silent G2G mutation were generated. (C) The plasmids were mixed at a 50:50 ratio before linearization in the sequence flanking both homology arms. (D) Integration was facilitated by CRISPR/Cas-9, and then following successful transfection, parasite genomic DNA was extracted. (E) Integration specific primers allowed a selective PCR amplification of the integrated fragment, to which MiSeq adapter sequences were attached. (F) The Illumina adapters were attached by ligation using the KAPA HyperPrep Kit. (G) To discriminate between samples, the adapter-ligated fragments were labeled with indices by indexing PCR with 2 indices at each end creating a unique barcode. This step also added the attachment site for the MiSeq instrument (P5 or P7). (H) Following Illumina sequencing, the ratio of the number of sequences for either the G2A or G2G variant provided an indication of the viability of each variant. The number of total reads with either the G2A or G2G sequence, for either 2 or 3 experiments, is indicated (see also S1 Data for the distribution of reads). ARO, armadillo domain–containing rhoptry protein; CDPK1, calcium-dependent protein kinase 1; GAP45, glideosome-associated protein 45; TRP, tetratricopeptide repeat protein. (PDF) [file pbio.3001408.s007.pdf]

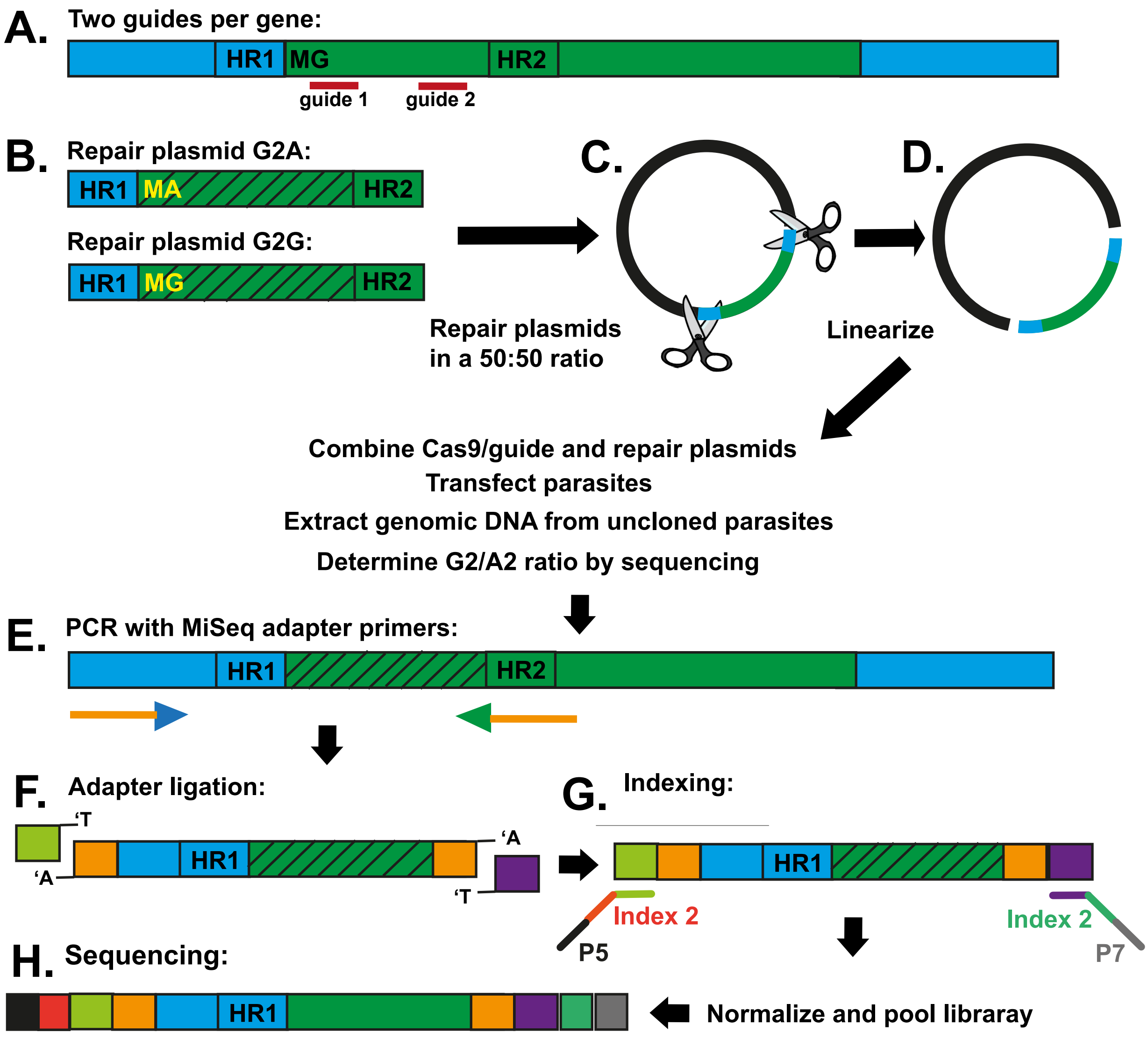

Reads	GAP45	ARO	CDPK1	S9C	TRP	ISP3
Expt. 1	2267	2012	43	1452	2327	660
Expt. 2	2184	2003	8	1809	2003	573
Expt. 3	2403					
