# Supplementary material for: Inhibition of protein N-myristoylation blocks Plasmodium falciparum intraerythrocytic development, egress and invasion
Source: PLoS Biol. 2021 Oct 25;19(10):e3001408. doi: 10.1371/journal.pbio.3001408 (PMC8544853; doi:10.1371/journal.pbio.3001408)
Supplement: S5 Fig — (A) The genetic complementation strategy used to introduce a second copy of the gap45 gene coding for either an N-terminal glycine (G2G) or alanine (G2A) and its promoter sequence into the pfs47 locus of a gap45:ha3:loxP parasite clone in the DiCre-expressing P. falciparum line B11, generating the gap45:ha3:loxP::comp_gap45[WT] (cGAP45[WT]) and gap45:ha3:loxP::comp_gap45[G2A] (cGAP45[G2A]) lines. (B) Schematic representation of the Pf47 locus with and without integration of the gap45 gene, and the oligonucleotide primers within the 5′ and 3′ UTR regions used to analyze integration by PCR. (C) PCR analysis of gap45:ha3:loxP::comp_gap45[WT] and gap45:ha3:loxP::comp_gap45[G2A] uncloned parasites showing 5′ and 3′ UTR integration. The B11 (3D7) line was used as a control for the WT parasite. (D) PCR analysis of 3 selected gap45:ha3:loxP::comp_gap45[G2A] clones. The B11 3D7 line was used as a control for the WT parasite. GAP45, glideosome-associated protein 45; WT, wild-type. (PDF) [file pbio.3001408.s008.pdf]

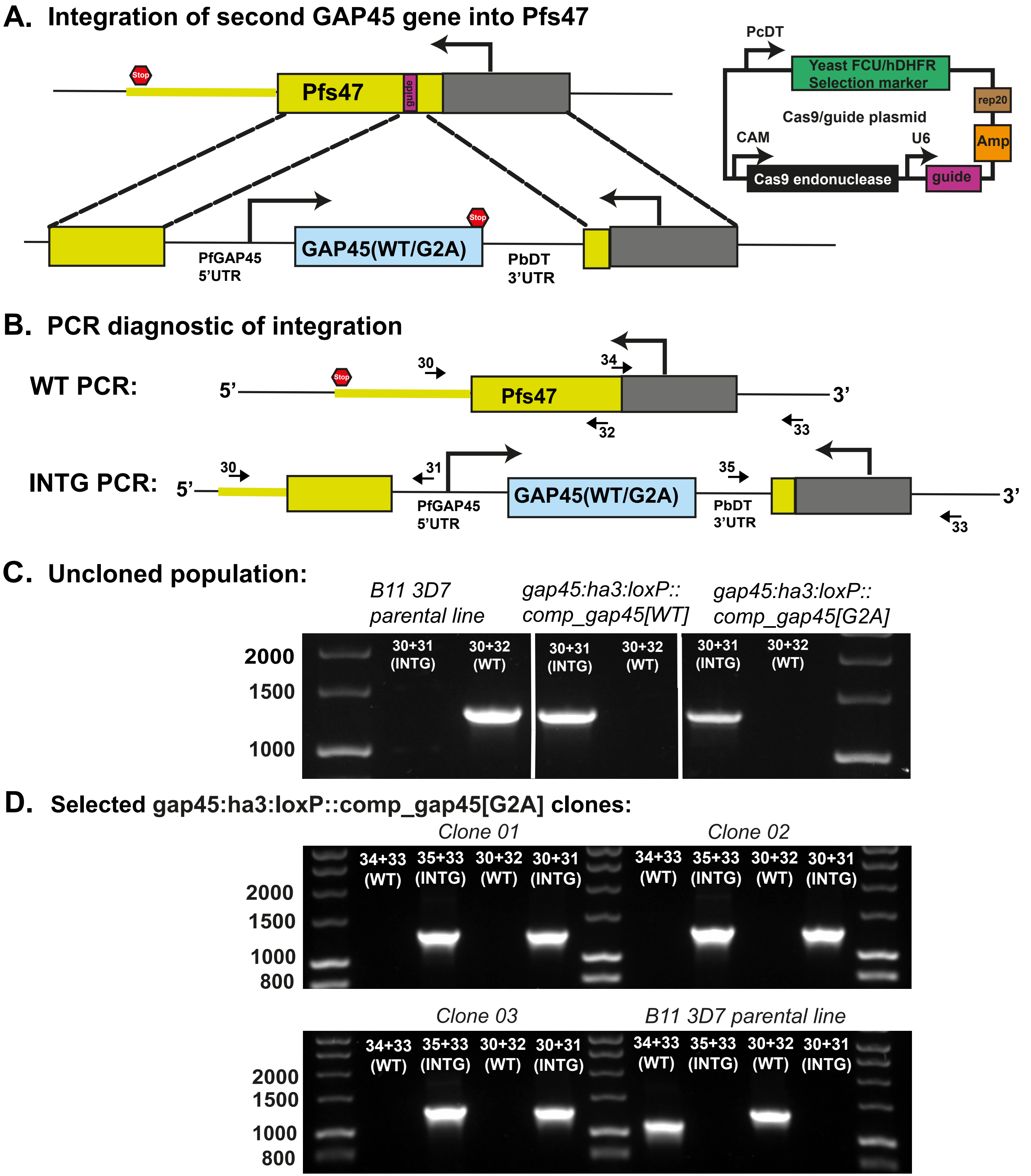
A. Integration of second GAP45 gene into Pfs47
Stop
Pfs47
guide
PcDT
Yeast FCU/hDHFR Selection marker
rep20
Cas9/guide plasmid
CAM
U6
Amp
Cas9 endonuclease
guide
PfGAP45 5'UTR
GAP45(WT/G2A)
Stop
PbDT 3'UTR
B. PCR diagnostic of integration
WT PCR:
5'
Stop
30
34
Pfs47
3'
32
33
INTG PCR:
5'
30
31
35
GAP45(WT/G2A)
3'
PfGAP45 5'UTR
PbDT 3'UTR
33
C. Uncloned population:
B11 3D7 parental line
gap45:ha3:loxP::comp_gap45[WT]
gap45:ha3:loxP::comp_gap45[G2A]
2000
1500
1000
30+31 (INTG)
30+32 (WT)
30+31 (INTG)
30+32 (WT)
30+31 (INTG)
30+32 (WT)
D. Selected gap45:ha3:loxP::comp_gap45[G2A] clones:
Clone 01
Clone 02
34+33 (WT)
35+33 (INTG)
30+32 (WT)
30+31 (INTG)
34+33 (WT)
35+33 (INTG)
30+32 (WT)
30+31 (INTG)
2000
1500
1000
800
Clone 03
B11 3D7 parental line
34+33 (WT)
35+33 (INTG)
30+32 (WT)
30+31 (INTG)
34+33 (WT)
35+33 (INTG)
30+32 (WT)
30+31 (INTG)
2000
1500
1000
800
