# Supplementary material for: Inhibition of protein N-myristoylation blocks Plasmodium falciparum intraerythrocytic development, egress and invasion
Source: PLoS Biol. 2021 Oct 25;19(10):e3001408. doi: 10.1371/journal.pbio.3001408 (PMC8544853; doi:10.1371/journal.pbio.3001408)
Supplement: S1 Raw Images — (PDF) [file pbio.3001408.s014.pdf]

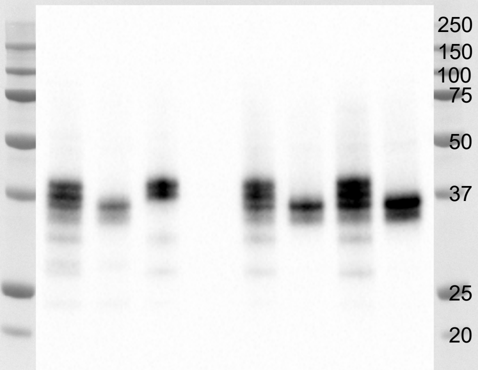

Fig 5A, Western blot of cell lysates with antibodies to GAP45, visualized with BioRad ChemiDoc system. The order of samples is as in Fig 5A. Molecular mass markers and their sizes (kDa) are superimposed.

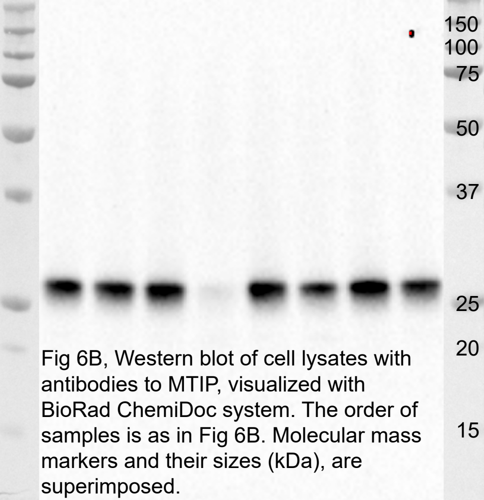

Fig 6B, Western blot of cell lysates with antibodies to MTIP, visualized with BioRad ChemiDoc system. The order of samples is as in Fig 6B. Molecular mass markers and their sizes (kDa), are superimposed.

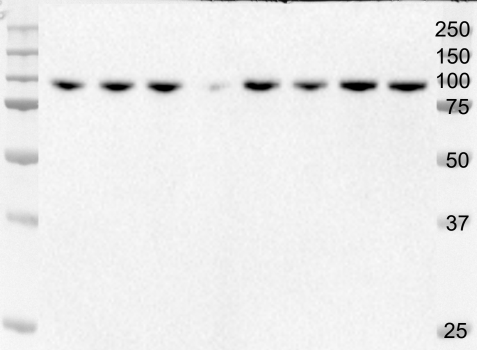

Fig 6B, Western blot of cell lysates with antibodies to MyoA, visualized with BioRad ChemiDoc system. The order of samples is as in Fig 6B. Molecular mass markers and their sizes (kDa), are superimposed.

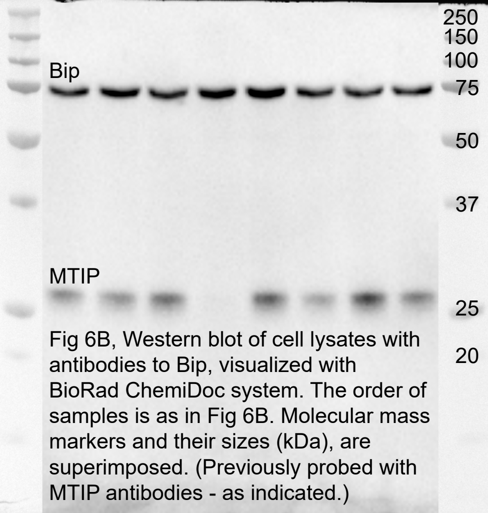

Fig 6B, Western blot of cell lysates with antibodies to Bip, visualized with BioRad ChemiDoc system. The order of samples is as in Fig 6B. Molecular mass markers and their sizes (kDa), are superimposed. (Previously probed with MTIP antibodies - as indicated.)

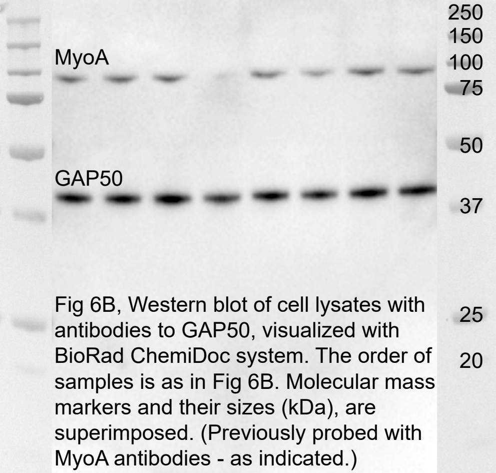

Fig 6B, Western blot of cell lysates with antibodies to GAP50, visualized with BioRad ChemiDoc system. The order of samples is as in Fig 6B. Molecular mass markers and their sizes (kDa), are superimposed. (Previously probed with MyoA antibodies - as indicated.)

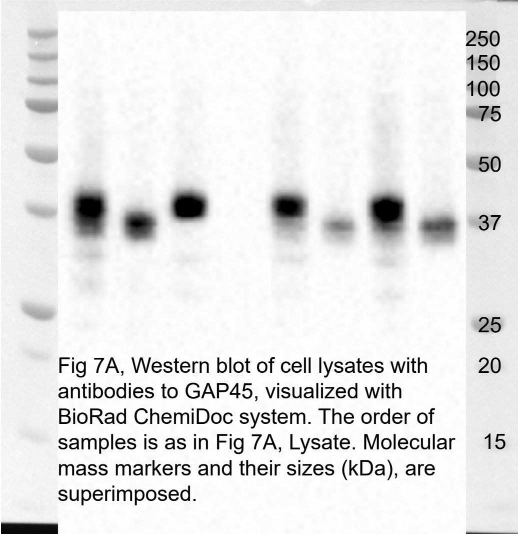

Fig 7A, Western blot of cell lysates with antibodies to GAP45, visualized with BioRad ChemiDoc system. The order of samples is as in Fig 7A, Lysate. Molecular mass markers and their sizes (kDa), are superimposed.

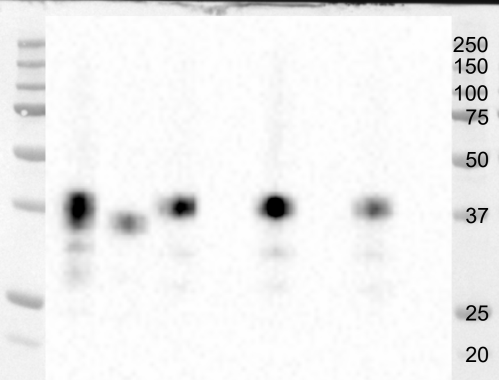

Fig 7A, Western blot of bound and eluted material with antibodies to GAP45, visualized with BioRad ChemiDoc system. The order of samples is as in Fig 7A, Bound and eluted. Molecular mass markers and their sizes (kDa), are superimposed.

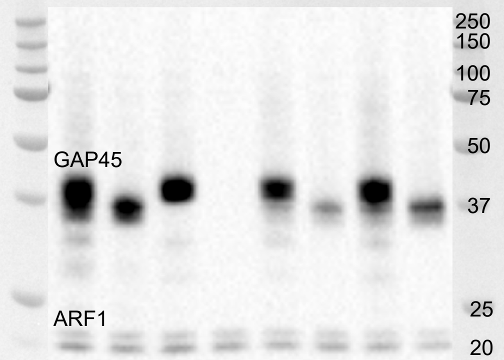

Fig 7A, Western blot of cell lysate with antibodies to ARF1, visualized with BioRad ChemiDoc system. The order of samples is as in Fig 7A, Lysate. Molecular mass markers and their sizes (kDa), are superimposed.(Previously probed with GAP45 antibodies - as indicated.)

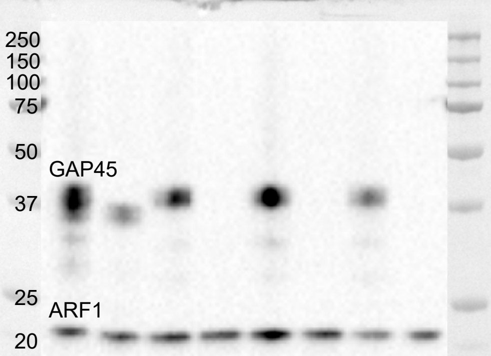

Fig 7A, Western blot of bound and eluted material with antibodies to ARF1, visualized with BioRad ChemiDoc system. The order of samples is as in Fig 7A, Bound and eluted. Molecular mass markers and their sizes (kDa), are superimposed.(Previously probed with GAP45 antibodies - as indicated.)

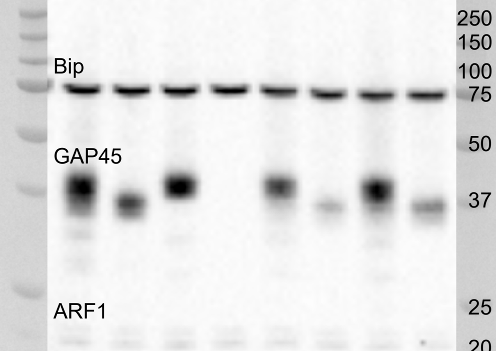

Fig 7A, Western blot of cell lysate with antibodies to Bip, visualized with BioRad ChemiDoc system. The order of samples is as in Fig 7A, Lysate. Molecular mass markers and their sizes (kDa), are superimposed.(Previously probed with GAP45 and ARF1 antibodies - as indicated.)

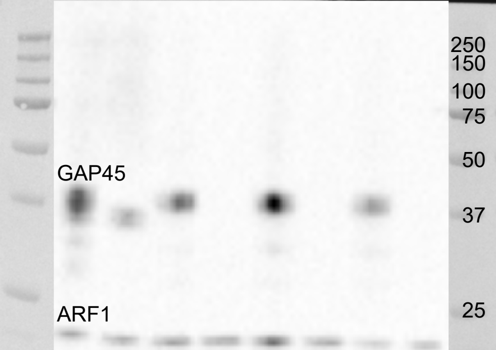

Fig 7A, Western blot of bound and eluted material with antibodies to Bip, visualized with BioRad Chemi Doc system. The order of samples is as in Fig 7A, Bound and eluted. Molecular mass markers and their sizes (kDa), are superimposed.(Previously probed with GAP45 and ARF1 antibodies.)

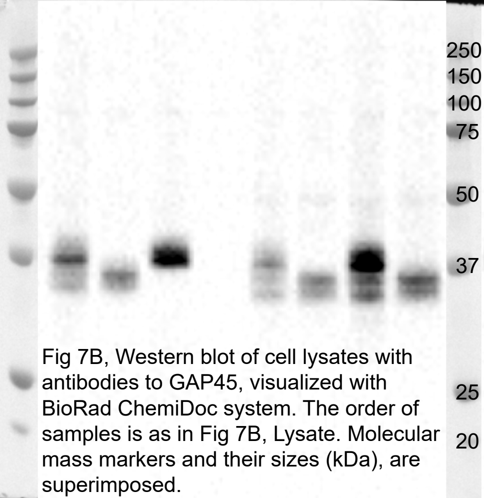

Fig 7B, Western blot of cell lysates with antibodies to GAP45, visualized with BioRad ChemiDoc system. The order of samples is as in Fig 7B, Lysate. Molecular mass markers and their sizes (kDa), are superimposed.

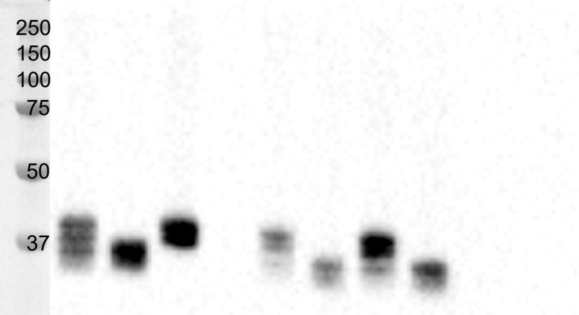

Fig 7B, Western blot of bound and eluted material with antibodies to GAP45, visualized with BioRad ChemiDoc system. The order of samples is as in Fig 7B, Bound and eluted. Molecular mass markers and their sizes (kDa), are superimposed.

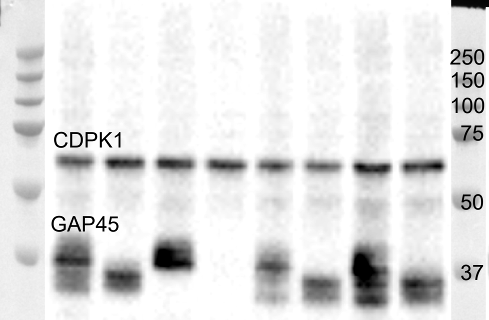

Fig 7B, Western blot of cell lysates with antibodies to CDPK1, visualized with BioRad ChemiDoc system. The order of samples is as in Fig 7B, Lysate. Molecular mass markers and their sizes (kDa), are superimposed. (Previously probed with GAP45 antibodies - as indicated.)

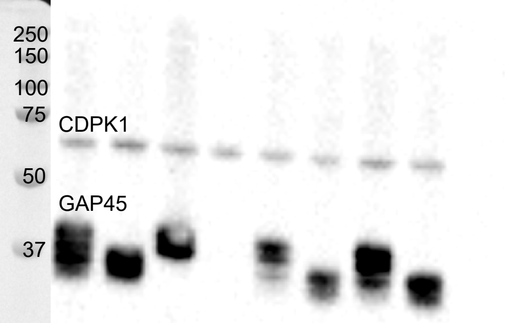

Fig 7B, Western blot of bound and eluted material with antibodies to CDPK1, visualized with BioRad ChemiDoc system. The order of samples is as in Fig 7B, Bound and eluted. Molecular mass markers and their sizes (kDa), are superimposed. (Previously probed with GAP45 antibodies - as indicated.)

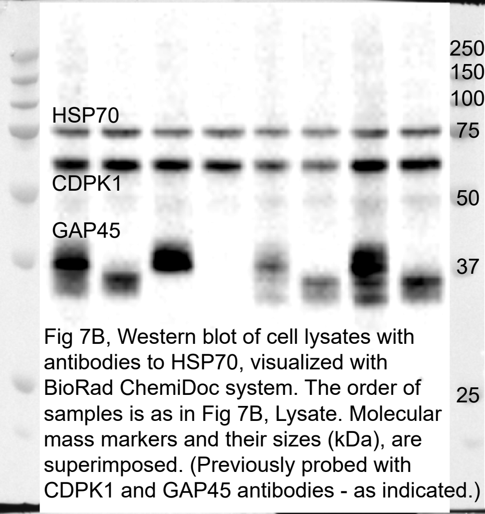

Fig 7B, Western blot of cell lysates with antibodies to HSP70, visualized with BioRad ChemiDoc system. The order of samples is as in Fig 7B, Lysate. Molecular mass markers and their sizes (kDa), are superimposed. (Previously probed with CDPK1 and GAP45 antibodies - as indicated.)

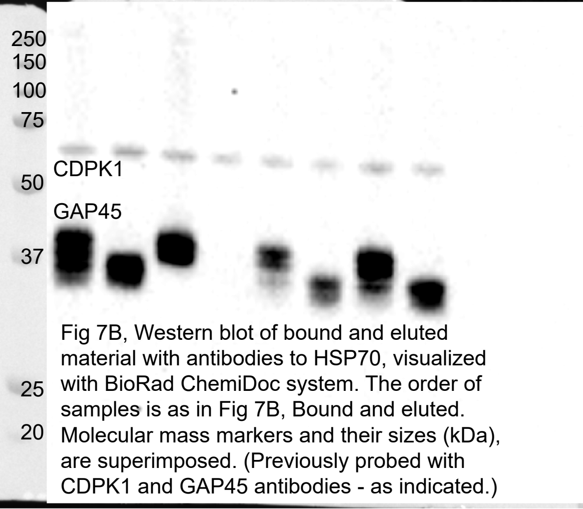

Fig 7B, Western blot of bound and eluted material with antibodies to HSP70, visualized with BioRad ChemiDoc system. The order of samples is as in Fig 7B, Bound and eluted. Molecular mass markers and their sizes (kDa), are superimposed. (Previously probed with CDPK1 and GAP45 antibodies - as indicated.)

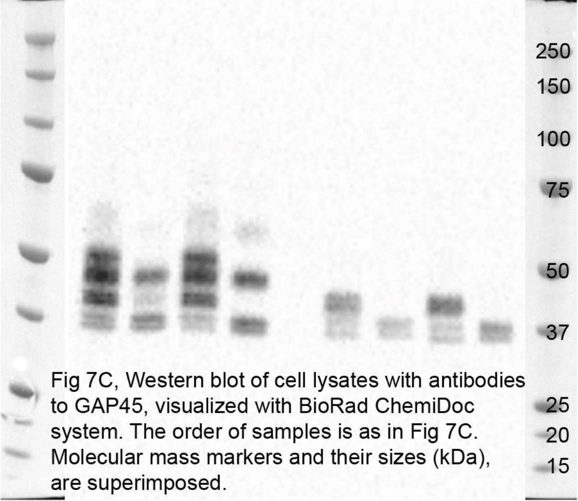

Fig 7C, Western blot of cell lysates with antibodies to GAP45, visualized with BioRad ChemiDoc system. The order of samples is as in Fig 7C. Molecular mass markers and their sizes (kDa), are superimposed.

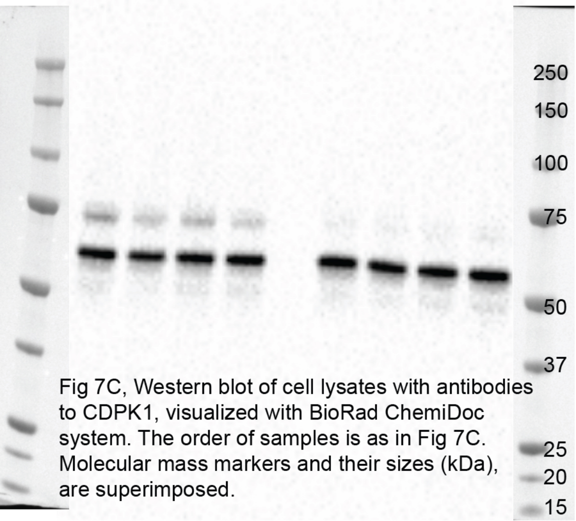

Fig 7C, Western blot of cell lysates with antibodies to CDPK1, visualized with BioRad ChemiDoc system. The order of samples is as in Fig 7C. Molecular mass markers and their sizes (kDa), are superimposed.

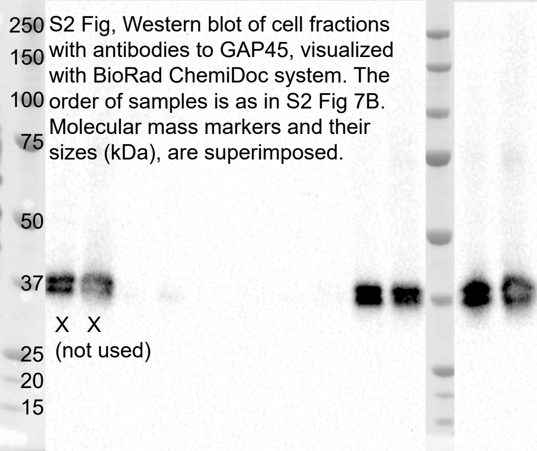

S2 Fig, Western blot of cell fractions with antibodies to GAP45, visualized with BioRad ChemiDoc system. The order of samples is as in S2 Fig 7B. Molecular mass markers and their sizes (kDa), are superimposed.

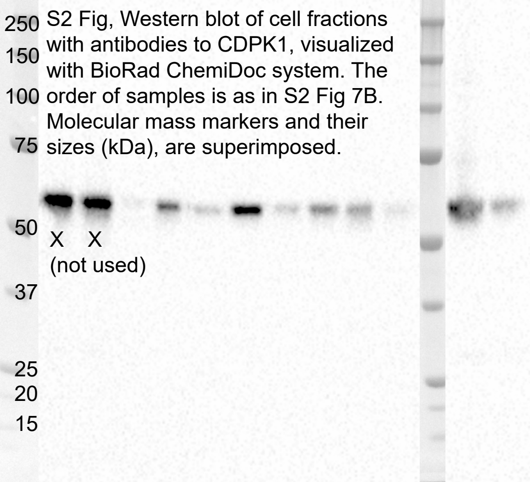

S2 Fig, Western blot of cell fractions with antibodies to CDPK1, visualized with BioRad ChemiDoc system. The order of samples is as in S2 Fig 7B. Molecular mass markers and their sizes (kDa), are superimposed.

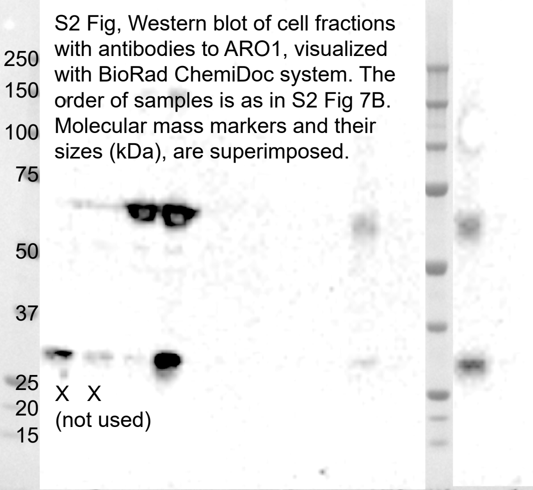

S2 Fig, Western blot of cell fractions with antibodies to ARO1, visualized with BioRad ChemiDoc system. The order of samples is as in S2 Fig 7B. Molecular mass markers and their sizes (kDa), are superimposed.

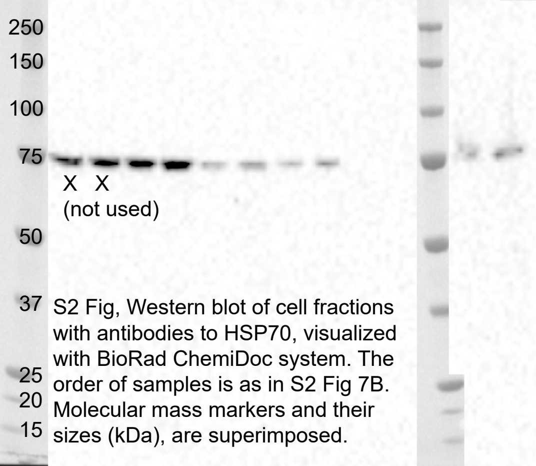

S2 Fig, Western blot of cell fractions with antibodies to HSP70, visualized with BioRad ChemiDoc system. The order of samples is as in S2 Fig 7B. Molecular mass markers and their sizes (kDa), are superimposed.

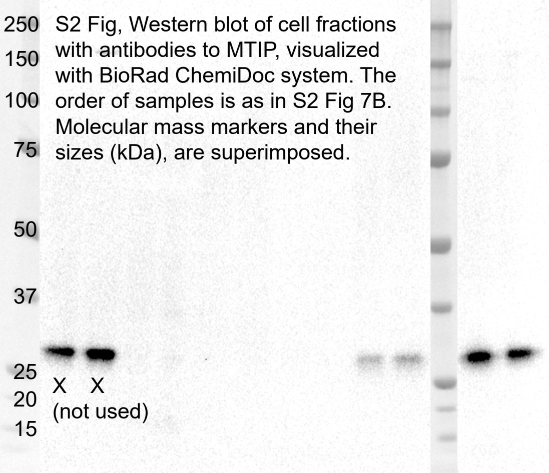

S2 Fig, Western blot of cell fractions with antibodies to MTIP, visualized with BioRad ChemiDoc system. The order of samples is as in S2 Fig 7B. Molecular mass markers and their sizes (kDa), are superimposed.

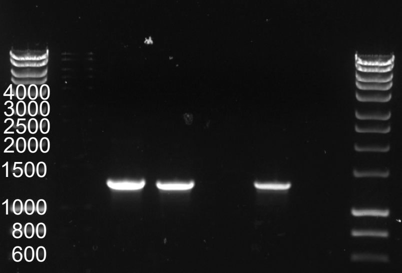

S5C Fig agarose gel analysis, sample order as in Fig. Size markers (bp) are indicated.

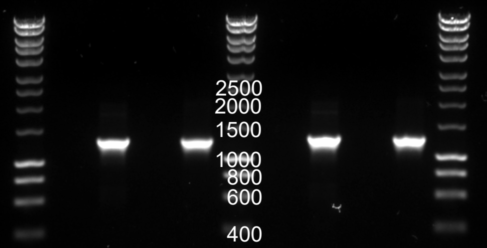

S5D Fig (top panel) agarose gel analysis, sample order as in Fig. Size markers (bp) are indicated.

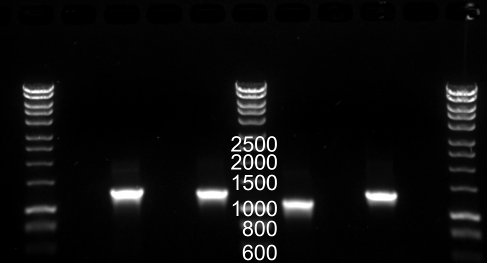

S5D Fig (lower panel) agarose gel analysis, sample order as in Fig. Size markers (bp) are indicated.

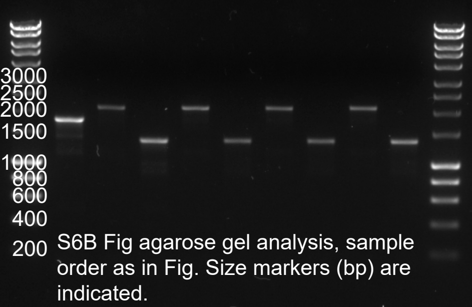

S6B Fig agarose gel analysis, sample order as in Fig. Size markers (bp) are indicated.

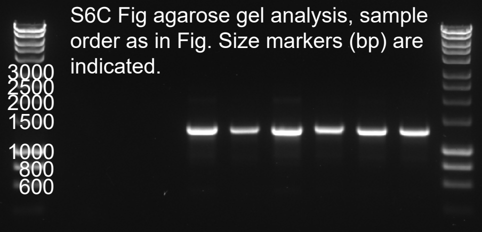

S6C Fig agarose gel analysis, sample order as in Fig. Size markers (bp) are indicated.
